# Phylogeny-driven pangenome analysis uncovers the genomic landscape of domesticated and wild Armeniaca species

**DOI:** 10.1093/hr/uhag104

**Published:** 2026-03-27

**Authors:** Ismael Blanchard, Quynh Trang Bui, Alexis Mergez, Sukanya Denni, Amandine Cornille, Isabelle Dufau, William Marande, Alexis Groppi, Stéphane Decroocq, Johann Confais, Ludovic Duvaux, Véronique Decroocq, Benjamin Linard

**Affiliations:** Université de Bordeaux, INRAE, UMR 1332 BFP, Département Biologie et Amélioration des Plantes (BAP), 71 Avenue Edouard Bourlaux, 33882 Villenave d'Ornon, France; Université de Bordeaux, INRAE, UMR 1332 BFP, Département Biologie et Amélioration des Plantes (BAP), 71 Avenue Edouard Bourlaux, 33882 Villenave d'Ornon, France; Université de Toulouse, INRAE, MIAT, UR 875, Département Mathématiques et Numérique (MathNum), Centre Occitanie-Toulouse, 24 Chemin de Borde Rouge, 31320 Auzeville-Tolosane, France; Université de Bordeaux, INRAE, BIOGECO, UMR 1202, Département Biodiversité Gènes et Communautés (BIOGECO), 69 Route d'Arcachon, F-33610 Cestas, France; Université de Rouen Normandie, UFR Sciences et Techniques, 3 Av. Pasteur, 76000 Rouen, France; Université Paris Saclay, INRAE, CNRS, AgroParisTech, GQE-IDEEV, 91190 Gif-sur-Yvette, France; New York University Abu Dhabi, Abu Dhabi, United Arab Emirates; Université de Toulouse, INRAE, Centre National de Ressources Génomiques Végétales-CNRGV, Département Biologie et Amélioration des Plantes (BAP), Centre Occitanie-Toulouse, 24 Chemin de Borde Rouge, 31320 Auzeville-Tolosane, France; Université de Toulouse, INRAE, Centre National de Ressources Génomiques Végétales-CNRGV, Département Biologie et Amélioration des Plantes (BAP), Centre Occitanie-Toulouse, 24 Chemin de Borde Rouge, 31320 Auzeville-Tolosane, France; Université de Bordeaux, Centre de Bioinformatique de Bordeaux (CBiB), 33076 Bordeaux, France; Université de Bordeaux, INRAE, UMR 1332 BFP, Département Biologie et Amélioration des Plantes (BAP), 71 Avenue Edouard Bourlaux, 33882 Villenave d'Ornon, France; Université Paris-Saclay, INRAE, URGI, US 1164, Departement Biologie et Amélioration des Plantes (BAP), BioinfOmics, 78026 Versailles, France; Université de Bordeaux, INRAE, BIOGECO, UMR 1202, Département Biodiversité Gènes et Communautés (BIOGECO), 69 Route d'Arcachon, F-33610 Cestas, France; Université de Bordeaux, INRAE, UMR 1332 BFP, Département Biologie et Amélioration des Plantes (BAP), 71 Avenue Edouard Bourlaux, 33882 Villenave d'Ornon, France; Université de Toulouse, INRAE, MIAT, UR 875, Département Mathématiques et Numérique (MathNum), Centre Occitanie-Toulouse, 24 Chemin de Borde Rouge, 31320 Auzeville-Tolosane, France

## Abstract

Long-read sequencing and pangenomics are revolutionizing crop research by providing more complete genome information and revealing crucial structural variations (SVs) linked to important agricultural traits. Building on recent advances in intraspecific pangenome construction, this study addresses the challenge of creating broader, cross-taxon pangenomes, using the Armeniaca taxonomic section as a model. Leveraging a diverse panel of genome assemblies as well as completing it with seven more genome assemblies generated for this study, we constructed a pangenome graph and cataloged the associated genetic variation, identifying approximately 25 million single nucleotide polymorphisms and over 537 000 structural variants. We characterized the diversity of these variants and assessed the extent to which different taxa contribute to overall pangenome expansion. Additionally, we evaluated the performance of low-depth sample mapping to the graph-based reference, highlighting key technical limitations that may affect the quality of downstream analyses. We further identified specific subsets of SVs that exhibit associations with particular classes of transposable elements (TEs). We showed that TEs are a major driver of SV, particularly insertions and deletions, with distinct size and distribution patterns (peaking in the 200- to 400-bp indel bin). They are also nonrandomly positioned in the genome, showing a tight concentration near coding genes, which suggests a role in gene regulation. As a case study illustrating the potential functional relevance of graph-derived SVs, we examined the genomic configuration of the Dormancy-Associated MADS box locus within the Armeniaca pangenome. These findings provide a framework to investigate adaptation in perennial fruit trees of the Armeniaca section.

## Introduction

Recent advances in long-read sequencing technologies have enabled the assembly of more complete and accurate plant genomes, advancing crop biology and breeding. However, single-reference assemblies represent only a fraction of genomic diversity, limiting our understanding of species-wide and taxa-wide genetic variation [1]. Pangenome studies often involve assembling genomes from multiple individuals from related and interfertile species, and reveal complex structural variations (SVs) that play a critical role in adaptation and domestication [2]. SVs affect gene content and repetitive elements, influencing agronomically important traits like stress resistance [3]. Despite challenges in computational infrastructure, emerging approaches aim to shift from single-reference genomes to pangenomes in crop improvement and ecological studies [4]. Most pangenome construction tools developed to date have been designed for intraspecific pangenomes [5]. Transitioning from species-level pangenomes to taxa-wide pangenomes presents a challenge, which we have addressed using the Armeniaca taxonomic section as a proof of concept.

Among the Armeniaca section, *Prunus armeniaca* L. (Rosaceae) [6] corresponds to both cultivated apricots and their wild forms, which are still growing in natural populations in Central Asia [7]. The initial domestication event occurred around 2900 years ago, originating from a natural population of southern Central Asian apricots and spreading toward China. A subsequent domestication event took place approximately 2200 years ago, originating from a northern Central Asian population and expanding into the Mediterranean basin [8]. Several other species within the genus *Prunus*, such as *P. sibirica* L.*, P. mandshurica,* and *P. mume,* are considered close relatives to the cultivated apricots [9].

The Armeniaca section is characterized by its high diversity and significant phenotypic variation, which has evolved since it diverged from *Amygdalus* species around 7 million years ago [8]. Although Armeniaca species are interfertile and possess collinear genomes, population studies based on a single reference genome often fail to capture critical phenotype-modulating genetic variants such as single nucleotide variants, structural variations (SVs), and copy number variations (CNVs). This limitation, known as reference bias, becomes more pronounced as genetic diversity increases, hindering the development of effective breeding strategies [10]. Pangenomic approaches have been introduced to overcome this challenge, with variation graphs representing genetic diversity across multiple genomes [10]. A pangenome aggregates all genetic variations, including single nucleotide polymorphisms (SNPs) and large SVs, within a species or species complex. This aggregation can extend beyond the species level to include closely related species, often referred to as super-pangenomes or meta-pangenomes [11]. Pangenome graphs have proven effective in characterizing intra- and interspecific genetic variability, enhancing the extraction and analysis of complex genetic traits [12]. Several plants of agronomic importance, including rice, cabbage, grapevine, and tomato, have been studied using pangenome graphs to capture complex SVs, some of which are restricted to wild types and absent in cultivars [11, 13, 14]. These studies have revealed valuable genetic diversity that can be used in breeding programs. Recently, the first *Prunus* pangenome graph was published for *P. persica* L., which identified key transposon variations linked to fruit coloration [15]. These advancements demonstrate the potential of pangenome graphs to uncover genetic traits that are vital for crop improvement and adaptation.

We present here an Armeniaca metapangenome comprising 25 chromosome-scale assemblies of three Armeniaca species, including European and Chinese common apricots (*P. armeniaca)*, Siberian apricots (*P. sibirica*), and Manchurian (*P. mandshurica*) apricots. SVs in the Armeniaca pangenome were identified using publicly available whole-genome short- and long-read data panels. The expanded variation catalog reveals structurally complex loci, and the pangenome’s accuracy was validated through the analysis of specific loci. We assessed the integration of new variations into the model, confirming its potential to enhance read mappings of novel sequence datasets. This approach reduces the bias associated with using a single genome as a reference. We characterized structural variants—primarily insertions and deletions (indels)—across different phylogenetic groups, and then uses this information, along with a pan-library of transposable elements (TEs), to analyze the TE landscape in relation to SV size and proximity to coding genes. Finally, variations related to a cluster of dormancy-associated genes (DAM genes) were explored, demonstrating the pangenome graph’s potential for future studies.

## Results

### Assemblies dataset

**Figure 1:**
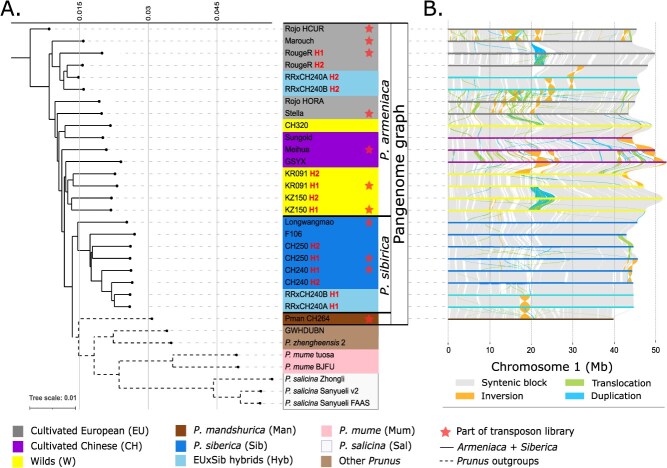
Evolutionary relationships among assemblies. **A**. Distance tree of selected Armeniaca genome assemblies. This tree aims to define an order for pangenome graph construction (see methods) and, as a consequence, is rooted on the selected reference genome Rojo_HCUR and not outgroups. Wherever possible, individual haplotypes are maintained as distinct leaves (labels H1 and H2). **B**. Global synteny among all of chromosome 1. Horizontal lines represent chromosome length, and colored vertical blocks represent all rearrangements detected between consecutive chromosome pairs.

For this study, we built a comprehensive dataset of 32 *Prunus* genome assemblies, each obtained from diverse sequencing technologies and research groups (Table 1). To facilitate the interpretability of our results, we categorized these assemblies into distinct groups based on their phylogenetic proximity, geographic origin, and agronomic utility (Table 1). This classification strategy also broadly aligns with the genetic groupings established by Ref. [8]. Our grouping scheme is as follows: the European (EU) group with assemblies of European origins, the wild (W) group representing wild Central Asian apricot genomes, the Chinese (CH) group with cultivated *P. armeniaca* genomes from China, the Siberian (Sib) group encompassing *P. sibirica* genomes along with one phylogenetically close *P. armeniaca* accession (Longwongmao), the Hybrid (HYB) group, which includes all *P. armeniaca x P. sibirica* F1 hybrids, and finally the Manchurian (Man) group, which includes the *P. mandshurica* genome (Pman_CH264). CH320, while being initially assigned to the Siberian apricots (Sib group), was shown to be a Central Asian type of *P. armeniaca* [8], which was confirmed here (Fig. 1).

**Table 1:** Genome assemblies used in this study and associated statistics

**Group**	**Assembly label**		**Haplotype**	**Species**	**Genetic cluster** ^**a**^	**Sequencing methods**	**BUSCO score (%)**	**Assembly size (Mb)**	**Assembly N50 (Mb)**
EU	Marouch		Unphased	*P. armeniaca*	C1	PB, SR	97.3	**204**	25.2^b^
	Rojo HORA		H1		C1	PB, HiC, 10x, SR	98.0	213	25.5
	Rojo HCUR		H2		C1	PB, HiC, 10x, SR	98.1	214	25.8
	Stella		Unphased		C1	PB, SR	97.9	209	25.5
	RougeR	★	H1		C1	PB-hi	**90.2**	211	28.9
	RougeR	★	H2		C1	PB-hi	98.1	238	28.5
CH	Sungold		Unphased	*P. armeniaca*	—	PB, HiC,BN	98.0	215	≤14.1^c^
	GSYX		Unphased		—	PB-hi	93.2	235	30.9
	Meihua		Unphased		—	PB,HiC, SR	97.8	238	30.1
W	KZ150	★	H1	*P. armeniaca*	W2	PB-hi, SR, BN	98.8	247	21.1
	KZ150	★	H2		W2	PB-hi, SR, BN	**99.0**	238	21.7
	KR091	★	H1		W1	PB-hi	96.7	233	28.5
	KR091	★	H2		W1	PB-hi	97.5	233	**29.7**
	CH320		Unphased		—	ONT	98.3	**259**	13.8^b^
Sib	Longwongmao		Unphased	*P. armeniaca*	—	PB, HiC,BN	98.2	218	≤14.1^c^
	CH240	★	H1	*P. sibirica*	W4	PB-hi	98.5	216	25.9
	CH240	★	H2		W4	PB-hi	98.6	214	25.3
	CH250	★	H1		W4	PB-hi, SR, BN	98.9	220	11.6
	CH250	★	H2		W4	PB-hi, SR, BN	98.5	213	**10.3**
	F106		Unphased		—	PB, HiC, BN	98.4	216	≤14.1^c^
Hyb	RRxCH240A	★	H1	*P. armeniaca × P. sibirica*	—	PB-hi	98.8	213	25
	RRxCH240A	★	H2			PB-hi	**99.0**	217	26
	RRxCH240B	★	H1			PB-hi	98.7	215	25.2
	RRxCH240B	★	H2			PB-hi	97.6	209	24.5
Man	Pman CH264		Unphased	*P. mandshurica*	*—*	PB, ONT, SR	94.7	224	14.5^b^

All assemblies marked with a star were *de novo* assembled for this study. Phased assemblies were split as H1/H2 because they can be represented as different paths in a pangenome graph. Extremes of BUSCO score, assembly length, and N50 are highlighted in bold. Sequencing methods: PB, PacBio; PB-hi, PacBio Hifi; HiC, Hi-C-based scaffolding; ONT, Oxford Nanopore; BN, BioNano; 10x, 10x scCNV; SR, short reads.

a Genetic clustering based on Ref. [8].

b As reported in Ref. [8]. Suppl. Data 3 and 4.

c As reported by the GDR assembly summary (see www.rosaceae.org/Analysis/10254124).

Seven assemblies (KZ150, RougeR, CH240, CH250, KR091, RRxCH240A, and RRxCH240B) were specifically *de novo* generated for the study (Table 1). The corresponding methods are detailed in Suppl. File S1, Section 1. Other assemblies were retrieved from previous studies or sequence databases (sources are listed in Suppl. Table T1). Notably, our dataset comprises both haplotype-resolved and unphased (mixed-haplotype) genomes (Table 1). To streamline our analysis, we will uniformly refer to each distinct input sequence, whether it represents a single haplotype or a mixed assembly, as an independent ‘assembly’. This approach is rooted in the operational mechanisms of pangenome graph tools, which interpret every input sequence as a unique ‘path’ within the graph. For example, the two resolved haplotypes of KZ150 (H1 and H2) will be represented as two independent ‘assemblies’ and, consequently, two distinct paths in the graph. Similarly, the unphased assembly of CH320 will be considered a single ‘assembly’, corresponding to one path in the graph. This standardization ensures consistent processing and interpretation across all diverse assembly inputs.

Among all assemblies, the Rojo_HCUR assembly [16] was selected as our ‘reference’ genome for this study (a prerequisite of the selected pangenome graph construction tools, see Methods). It originates from the *Rojo* F₁ hybrid between ‘Currot’ and ‘Orange Red’, for which two high-quality, chromosome-level, haplotype-resolved assemblies were produced using a technique called gamete binning. This method leverages high-throughput single-cell sequencing of pollen grains from the hybrid, enabling the separation of reads corresponding to each parental haplotype. As a result, two haplotype-specific assemblies Rojo_HCUR (representing the Currot-derived haplotype) and Rojo_HORA (representing the Orange Red-derived haplotype) were obtained. Each assembly was scaffolded at the chromosome level and exhibited over 99% haplotyping precision and alignment accuracy with their respective parental genomes. The selection of such high-quality tail-to-tail haplotype as a reference will ensure a higher quality reference for future population-level analyses and association studies.

### Assemblies curation and preliminary steps for graph construction

All retrieved assemblies underwent the same steps of curation (see Methods). BUSCO scores against the Eudicot_do10 gene set are all above 95%, except for RougeR H1 (90.2%) and GSYX (93.2%). Genome lengths range from 204 Mb (EU group, Marouch) to 259 Mb (W group, CH320). Being based on different long-read technologies (Table 1), all assemblies show high N50 values, ranging from 10.3 to 29.7 Mb. General statistics for all assemblies are reported in Suppl. Table T5. Chromosome synteny was consistent across the Sib and W groups, as illustrated in Fig. 1B for chromosome 1, chromosomes 2 to 8 in Suppl. File S1, Section 1; Fig. S1. Within the European (EU) and Chinese (CH) haplotype groups, a subset of haplotypes exhibited substantial structural variations (SVs)—specifically, inversions and duplications—when compared to the other members of their respective groups.

Namely, the two haplotypes, Rojo_HCUR and Rojo_HORA, displayed a higher frequency of short inversions and translocations compared to other EU assemblies. A similar pattern of structural variations was observed for GSYX within the CH group, even following the implementation of our previously described corrections for chromosome orientation. These distinct patterns of inflated structural variation were evident across the majority of the chromosomes analyzed (Suppl. File S1, Section 2; Table S1). Four assemblies showed pervasive inconsistencies in chromosomal k-mer distances and sizes for all of their chromosomes, indicating a lack of harmonized chromosome labeling. This necessitated the relabeling of corresponding sequences, a process thoroughly detailed in Suppl. File S1, Section 3; Fig. S2. In addition, the GSYX assembly showed multiple instances of reversed sequence orientations, which were subsequently corrected manually (Suppl. File S1, Section 3; Fig. S3).

Finally, a comprehensive phylogeny of all corrected assemblies was built to validate evolutionary relationships among the curated assemblies and to compare it with a previous one obtained from SNP [8]. To complement this analysis, we introduced seven outgroup assemblies, which were grouped into three categories (Mum for *P. mume*, Sal for *P. salicina*, Other for interspecific genomes); they are indicated by dashed branches in Fig. 1A. This resulting phylogenetic tree reveals several noteworthy placements. As expected, haplotypes in hybrid assemblies segregate according to their parental origin (e.g., RRxCH240 H2 within the European group and RRxCH240 H1 within the Siberian group, as illustrated in Fig. 1A). Second, the phylogenetic position of Meihua and GSYX (both from the Chinese group) makes the Wild group non-monophyletic. Lastly, although classified as *P. armeniaca* in the Genome Database for Rosaceae (GDR, www.rosaceae.org), ‘Longwongmao’ is phylogenetically basal to the *P. sibirica* lineage. This result coincidentally confirms the large genetic distance gap between the assemblies retained for graph construction and the outgroups (Suppl. File S1, Section 4; Fig. S4). It also motivated the decision to include only the *P. mandshurica* assembly as the sole outgroup during the process of pangenome graph construction and was primarily driven by the understanding that pangenome graph tools are currently designed for species-centric analyses and may yield suboptimal alignments when incorporating distantly related genomes, which could impact graph quality and could generate erroneous genetic variants artifacts. Consequently, our final dataset for graph construction comprised 25 assemblies (16 phased and 9 unphased, including one outgroup). The resulting tree is almost identical to the full tree, including all seven outgroups (Suppl. File S1, Section 5; Fig. S5), and was subsequently utilized as the guiding tree input for pangenome graph construction.

### The Armeniaca pangenome graph

Our pangenome graphs, constructed for each chromosome, exhibited a substantial complexity (see statistics in Suppl. File S1, Section 6; Table S2). The number of nodes per graph varied from 2.7 million to 6.7 million—with an average node length ranging from 27 to 41 base pairs—indicating SV occurrences every few dozen of base pairs, approximately. As expected, the largest chromosomes contained the highest number of nodes (e.g., chromosome 1 with 6 669 924), while the smallest, chromosome 5, had only 2 697 166 nodes. The compression factor, defined as the ratio between the total sequence length of all input assemblies and the total number of base pairs in the graph’s nodes, ranged from 4.01 (chromosome 2) to 6.38 (chromosome 5), reflecting differences in sequence redundancy and local structural complexity across the chromosomes. The Pansel analysis of our pangenome graphs (Suppl. File S1, Section 6; Table S2) revealed a varied distribution of significantly conserved and divergent regions (under Pansel definition) across chromosomes. The number of conserved regions ranged from 124 on chromosome 8 to 296 on chromosome 1. Similarly, the number of divergent regions varied from 579 to 1524, also across chromosomes 8 and 1, respectively.

**Figure 2:**
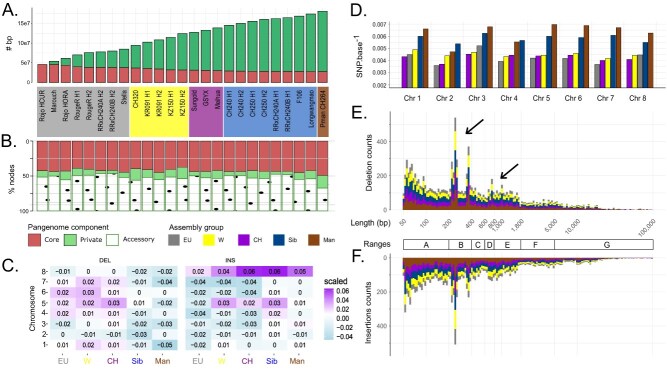
Pangenome variant diversity. **A.** Assembly contribution to pangenome growth (e.g., chromosome 1). The histogram quantifies the increase in pangenome content, measured in added base pairs with each newly added assembly (from left to right). Assemblies and their associated groups are delineated along the x axis, with distinct color coding (see legend). Red bars represent the core genome bases, defined as sequences shared by all analyzed assemblies. Green bars denote the total number of bases present in at least one assembly, encompassing the core, accessory, and private genome components, as per the definitions employed by the Panacus tool. **B.** Individual assembly composition per pangenome component (e.g., chromosome 1). Red fraction represents the core genome, comprising graph nodes shared by all assemblies; dotted fraction shows the accessory genome, encompassing graph nodes shared by more than two assemblies; green fraction indicates the private genome, consisting of graph nodes unique to a specific assembly. **C.** Normalized number of SV base pairs relative to Rojo_HCUR. For each group and chromosome pair, sums of SV lengths were corrected by chromosome lengths. Then all normalized total lengths were mean centered to allow direct comparison between groups and chromosomes. The gradient highlights the groups and chromosomes that are relatively depleted (clear blue) or enriched (purple) in inserted or deleted bases. **D.** SNP contribution per chromosome and assembly group. **E.** Distribution of deletion lengths on chromosomes. The x axis, on a log10 scale, represents the sorted lower bounds of indel length intervals (bins). The y axis stacks the mean number of indels observed within each assembly group. Arrows point out some of the indel peaks that motivate further analysis of transposon enrichments in some length ranges. **F.** An equivalent representation of E, but for insertions. All deletions/insertions are relative to the Rojo_HCUR reference genome.

The various assemblies and their respective phylogenetic groups contributed differentially to the genetic variations observed within the pangenome graph. Using the graph of (the longest) chromosome 1 as an example (other chromosomes in Suppl. Table T2), the incorporation of additional assemblies led to a reduction of the core pangenome and an expansion of its other components (Fig. 2A). Focusing on the European (EU) group, we observed an initial increase in total genomic content from 45.5 to 84.6 Mb, followed by a plateau. This saturation suggests that adding more assemblies may not significantly introduce novel genomic variations. Concurrently, within the same group, the core genome declined from 45.5 to 37.8 Mb, stabilizing across the RRxCH240 to Stella additions. This observation further reinforces previous findings regarding the core genome’s stability within the same lineage [17]. Then, the progressive integration of assemblies from various geographic origins significantly influenced pangenome size and core genome composition (Fig. 2A). The addition of Wild (W) accessions resulted in a substantial expansion of the pangenome, increasing its total size from approximately 94.2 to 124.0 Mb, representing an average gain of ~8 Mb per genome. Concurrently, the core genome observed a contraction from 37.7 to 31.6 Mb. Subsequent incorporation of Chinese (CH) assemblies continued this expansion, with the core genome further diminished to 29.6 Mb. The addition of Sibirica (Sib) genotypes resulted in a more modest pangenome expansion, contributing approximately ~4.3 Mb per genome. It is noteworthy that the RRxCH240A and RRxCH240B haplotypes contributed minimally to the overall pangenome increase, likely due to their hybrid nature derived from parental assemblies (RR and CH240) previously incorporated into earlier graph iterations. Finally, the Manchurian assembly contributed an almost exclusively accessory fraction of approximately ~7.2 Mb. The contribution of each assembly to the pangenome was further analyzed by quantifying the proportional distribution of core, accessory, and private graph nodes (under Pan1c tool definition) (Fig. 2B). Core contributions ranging from 37% to 49% may be surprising but is attributable to the representation of repeated sequence patterns within the graph; a single node representing a repeat can be traversed multiple times by an assembly’s path, leading to repeated counting. Therefore, a higher core fraction in this representation may indicate potential duplications of core pangenome segments or ubiquitous genome repeats with variable copy numbers across assemblies. In contrast, contribution from the assemblies to the private and accessory nodes showed high variability, reaching up to 18% for *P. mandshurica*. Notably, the wild (W) group provided a substantial number of private nodes. Analyses of other chromosomes (Suppl. Table T3) revealed significant variations in the proportions of core, private, and accessory genomes. For example, on chromosome 3, core fractions ranged from 17% to 38%, with accessory content dominating in certain haplotypes (e.g., CH_320, ~49%). Conversely, the core genome exceeded 50% for some assemblies on chromosome 5, while private fractions spanned from negligible (<1%) to substantial (>30%). These diverse profiles underscore the chromosome-specific and lineage-specific nature of shared genomic variations within our pangenome.

### Structural variations within the pangenome

Our pangenome graph revealed significant genetic diversity across accessions. We observed a total of 25 million SNPs relative to the reference Rojo_HCUR (Suppl. Table  T4). SNP counts ranged from 1.4 million for Pman_CH264 to over a total of 10 million for the Sibirica group. While most groups showed consistent SNP density per assembly (Fig. 2D), it significantly increased for the Sibirica and Manchurian groups, reflecting their divergences with the EU, CH, and W groups. Chromosomes 2 and 4 consistently showed lower SNP contributions, suggesting stronger evolutionary constraint (Suppl. Table  T4).

The structural variants extracted from the graph (relatively to the reference Rojo_HCUR) showed complex distributions. The graph also contained over 537 000 indels (Suppl. Table  T4). Since indels (insertions and deletions) are defined relative to the Rojo_HCUR reference genome, their frequency is strongly correlated with phylogenetic distance. This relationship is quantitatively supported by the normalized counts of insertion and deletion base pairs in Fig. 2C. When analyzed from a phylogenetic group perspective, the indel distribution aligns with expectation under a domestication scenario, i.e., insertions (relative to Rojo_HCUR) are enriched in noncultivated groups and deletions are conversely depleted in noncultivated groups. However, these patterns exhibit greater complexity when examined at the chromosome level. For example, the noncultivated Wild (W) and Sibirica (Sib) groups demonstrated a significantly higher count of insertions on chromosomes 5 and 8 compared to the cultivated European (EU) and Chinese (CH) groups (Fig. 2C). When grouped and sorted by length bins (Fig. 2E and F), the smallest bin (50–200 bp) accounted for 40% of all pangenome indels while the largest bin (5000–100 000 bp) accounted for only 7.5%. Notably, two specific bins (200–400 bp and 800–1800 bp) displayed distinct peaks in indel counts across all chromosomes and phylogenetic groups (details for all bins in Suppl. Table  T4).

### Gene and TE landscape within the pangenome

**Figure 3:**
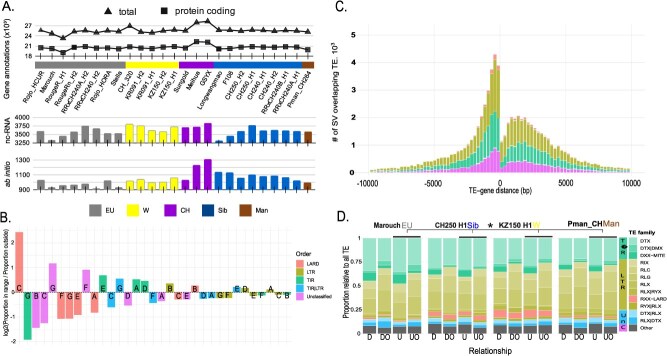
Genomic content, noncoding RNAs (ncRNA), and TEs distribution across the pangenome. **A.** Gene and noncoding RNA content. Panel A shows the total number of gene models (y axis) for each individual assembly (x axis), including subsets for ncRNAs and *de novo* predicted genes. This highlights the overall gene content and the distribution of these specific elements across phylogenetic groups. **B**. TE enrichment/depletion across indel (insertion and deletion) lengths. Panel B displays the enrichment (positive values) and depletion (negative values) patterns of TE orders (e.g., LTR, TIR) across various indel length bins. Associations are ordered from left to right based on the absolute magnitude of enrichment or depletion value, identifying the most divergent (on the far left) relationships between TE orders and indel sizes. Letters correspond to indel length intervals (bins) as depicted in Fig. 2E, going from A (50–200 bp) to G (5–100 kbp). **C**. Distance distribution between the TE-associated structural variants and their nearest coding genes. Panel C visualizes the distribution of genomic distances (on a log10 scale, x axis) between TE-associated SVs and their closest coding genes. Distances are stratified by TE order/family (e.g., LTR, LARD) and positional context (upstream vs downstream). The stacked distributions show the frequency of each TE order at a given distance, revealing order-specific biases in TE-gene proximity. **D**. Global TE family composition by haplotype. Panel D presents the proportional contribution of each TE family within SVs that overlap a TE for the four focal haplotypes (Marouch, CH250 H1, KZ150 H1, Pman_CH264). Proportions are shown for upstream and downstream positional categories relative to the nearest coding gene as described in Ref. [18], with D corresponding to downstream, DO to downstream overlap, U to upstream, and UO to upstream overlap. Statistical significance in Fig. 3D refers to differences between haplotypes in their upstream TE-order composition (see Suppl. Table T6: Transposon Distribution and Enrichment Analysis.xlsx).

Annotation of all assemblies revealed a variable total gene count, ranging from 23 318 to 28 360 genes (Fig. 3A). The GSYX and Meihua assemblies exhibited higher proportions of duplicated gene models, registering 12% and 10%, respectively, compared to less than 4% for remaining assemblies. When restricting the analysis only to assemblies with BUSCO scores exceeding 95% (Table 1), the gene content proved highly stable, displaying a mean of 25 650 genes (standard deviation: 680) (Fig. 3). Among these annotations, the number of noncoding RNAs was variable and showed no correlation with genome length, phylogenetic groups, or sequencing technologies. Detailed annotation statistics per assembly, including ignored pseudogenes, are provided in Suppl. Table T5.

The analysis of the transposable landscape was conducted by cross-referencing an assembly-centric transposon annotation with indels that were identified using a graph-based annotation approach, alongside preexisting or *de novo* gene annotations (Fig. 3A; Suppl. File S1). The aim was here to contextualize the transposon landscape in terms of gene proximity, phylogenetic context, and associated indel characteristics. To characterize TE dynamics within our pangenome, we first built a pan-library of 19 136 REPET consensus elements, refined to 8032 nonredundant definitions (Suppl. Data D1). Using this library, we detected 42 679 TEs within graph-based indels (≈1 TE per 18 kb). TE abundance varied strongly with indel size, peaking in the 200- to 400-bp bin and reaching a minimum in the 600- to 800-bp range (Suppl. File S1, Section 9; Fig. S7; Suppl. Table T6). TE orders showed marked size-dependent patterns: LARD elements were enriched in 400- to 600-bp indels (>30%), LTR elements remained consistently abundant (>20%), and unclassified TEs increased sharply beyond 1.8 kb. TIR elements were depleted from indels >5 kb (Fig. 3B).

To refine these library-wide patterns in a haplotype-specific context, we analyzed four representative assemblies (i.e., Marouch as a representative of the EU group, KZ150 H1 for the wild *P. armeniaca*, CH250 H1 for *P. sibirica*, and Pman_CH264 for *P. mandshurica*) and intersected their TE annotations with SV intervals (Fig. 3C). The distances between TEs and their nearest coding genes exhibited a broad range, spanning from −51.7 to +93.1 kb, yet demonstrated a pronounced spatial concentration in proximity to coding regions. Approximately 75% of the TEs inserted within ±2.6 kb of a start codon, and a significant depletion was observed precisely at the 0-bp position (∼500 fewer events), indicating that the random insertion of TE in very close proximity to the gene start might exert a deleterious effect on the adjacent gene, resulting in counter-selection of such events.

TE family composition was highly consistent across haplotypes (Fig. 3D). DTX elements dominated both upstream (~31%) and downstream (~30%), while LTR families RLG (class I retrotransposon Gypsy) and RLC (class I retrotransposon Copia) were stable across contexts (15–16% and 13–14%). Minor families (DXX-MITE, RIX, LARD) contributed 5%–9% with minimal interhaplotype variation (<5%). Upstream and downstream profiles were nearly identical, indicating no directional bias in TE usage relative to gene orientation. In contrast, chi-square analyses revealed strong haplotype-specific differences in TE-order composition (Suppl. File S1, Fig. S8).

### Enhanced mapping with graph-based approaches

Read mapping performance varied considerably depending on the specific tools employed and the methodologies used for projecting to genomes (e.g., how BAM files were generated). Notably, differences in postmapping filtering capabilities were software dependent (a detailed discussion on this is available in Suppl. File S1, Section 10; Fig. S9). Specifically, we found that ‘proper mapping’ or ‘paired segments’ flags associated with output BAM files are not directly comparable between current Variation Graph (VG) tools and Minimap2. VG tools appeared to employ a more stringent definition of proper mapping compared to Minimap2. Concurrently, analysis of MAPQ score extraction from all mapped samples confirmed that the corresponding scoring scheme is software specific, invalidating direct comparisons based on MAPQ values. Furthermore, MAPQ distributions generated by Minimap2 and VG Giraffe exhibited substantial differences (Suppl. File S1, Section 11; Fig. S10). Despite these limitations and to provide an approximate overview of mapping quality variations, we categorized corresponding reads into MAPQ extremes (Table 2).

**Table 2:** Read counts and proportion of reads associated with MAPQ score extremes.

MAPQ bin	Minimap2, Rojo_HCUR ref	VG Giraffe, pangenome graph
	Count (% of total)	Count (% of total)
≤5 (lowest)	3.71 (31.7)	**5.07 (43.3)**
Other values	1.14 (9.7)	**1.33 (11.3)**
≥55 (highest)	**5.59 (47.8)**	4.34 (37.1)
Filtered out	**1.27 (10.8)**	0.97 (8.3)

Following the mapping of the 322 short-read samples, reads were classified based on their associated MAPQ. These scores in [0;60] and scores <5 and > 55 are associated with a low-quality mapping or a high-quality mapping, respectively (1st column). The line ‘filtered out’ corresponds to reads that did not pass the samtools filters preceding mapping, and that are not associated with a MAPQ value. For each pair of tool and MAPQ bin, the left value reports the absolute number of reads (in milliards of reads) and the right value (in parentheses) reports the corresponding proportion among the total of reads. Highest and lowest values are highlighted in bold.

**Figure 4:**
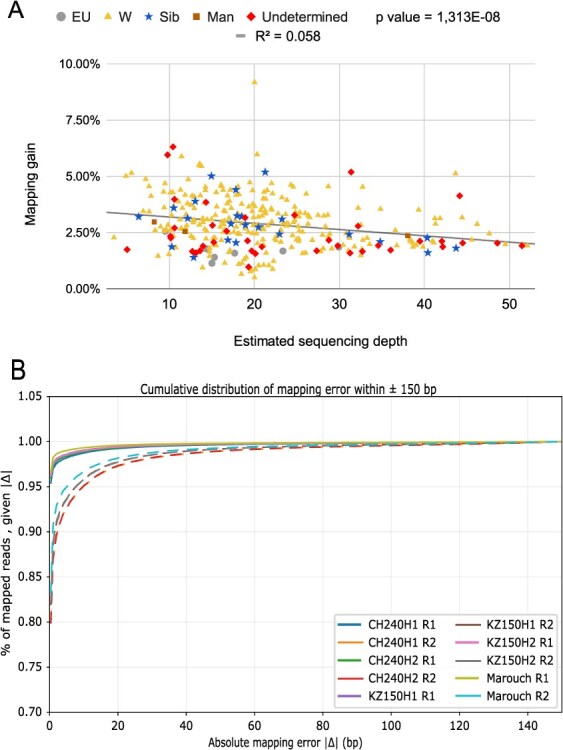
Pangenome graph enhances read mapping for low-depth accessions. **A.** This analysis quantifies the mapping gain observed for 322 low-depth short-read accessions when mapped to a pangenome graph, as opposed to a traditional single-reference mapping approach using Rojo_HCUR. The x axis represents the total number of reads generated for each accession, while the y axis displays the percentage of supplementary reads successfully mapped to the pangenome graph that were not mapped to the Rojo_HCUR reference. The gray line illustrates the linear regression between these two variables, revealing a weak negative correlation between sequencing depth and mapping gains. This analysis quantifies the mapping gain achieved by aligning 322 short-read accessions to the Armeniaca pangenome graph compared to a single-reference assembly (Rojo_HCUR). The x axis represents the normalized sequencing depth for each accession, obtained by dividing the total number of sequenced bases by the average haploid genome size of its corresponding genetic group. The y axis displays the percentage of supplementary reads successfully mapped to the pangenome graph that were not mapped to the Rojo_HCUR reference. The gray line illustrates the linear regression between these two variables, revealing a modest but statistically robust negative correlation between the normalized sequencing depth and the observed mapping gain**.** The results demonstrated a mapping gain ranging from 0.5% to 9.2% of supplementary reads successfully aligned to the graph. **B.** Positional shift of simulated paired-end reads when remapped to haplotype-depleted pangenome graphs. The simulation was run for three accessions: Marouch (EU group), CH240 (W group), KZ150 (Sib group). On the x axis, |Δ| represents the absolute shift (in base pairs) between the observed 5′ reference coordinate after mapping and the origin coordinate of the simulated read. The y axis represents the conditional cumulative fraction of reads with |Δ| ≤ x. Under this conditional formulation, all curves converge to 1 by definition.

This analysis revealed that Minimap2 yielded a higher proportion of reads with high MAPQ scores, while graph-based mappings generally had lower MAPQ scores but mapped more reads overall (Table 2). To refine this picture, Fig. 4A reports mapping gains when using the graph, e.g., the proportion of reads that could be mapped on the graph, but not when using the single reference assembly Rojo_HCUR. Crucially, graph-based approaches demonstrated enhanced mapping. All 322 short-read samples showed improved mapping to the pangenome graph, ranging from 0.5% to an outlier of 9.2% increased mapped reads compared to a single reference. A weak inverse correlation was observed: lower sequencing depths yielded higher gains, while samples with over 60 million reads converged to a stable 2.4% gain.

To quantify the interest of the Armeniaca pangenome graph, Fig. 4A illustrates the mapping gain, which refers to the proportion of sequenced reads that successfully aligned to the pangenome graph but failed to align to the single-reference assembly, specifically Rojo_HCUR. Here, graph-based mapping methodologies demonstrated a superior alignment capacity. All 322 high-quality short-read samples exhibited improved mapping efficiency when utilizing the pangenome graph. The observed mapping gains across the samples ranged from a minimum of 0.5% up to an exceptional outlier value of 9.2%. The estimated sequencing depths spanned from 3.5× to 52.1×, with a mean depth of 21.3×. A weak, inverse correlation was identified between the sequencing depth and the mapping gain (*R*^2^ = 0.058) with a *P* value <1.3 × 10^−7^ (*F*-test of *R*^2^). This indicates that samples displaying lower sequencing depth tended to yield higher mapping gains.

To further assess the positional accuracy of graph-based mapping, we used a leave-on-out approach inspired by simulations in Ref. [19]. Reads were simulated from three selected haplotypes in EU, CH, and Sib groups and remapped to a graph depleted from the corresponding path (see section Methods). Notably, most simulated reads were successfully remapped to some position of the pangenome graph, leading to an average recall of 97.1% (details in Suppl. File S1, Section 12). To assess precision in more detail, we quantified positional accuracy of remapped reads by measuring the absolute shift Δ between the true origin coordinate of simulated read mates and the observed coordinate on the depleted graphs. Figure 4B illustrates the conditional cumulative distribution of this shift. Exact coordinate recovery (|Δ| = 0) follows a consistent gradient with phylogenetic proximity to our reference, from Marouch (EU), KZ150 (W) to CH240 (Sib). For forward read mates (R1), exact placement rates ranged from 95.42% for CH240 to 96.66% for Marouch. Lower rates from 79.84% (CH240) to 83.20% (Marouch) were observed for the reverse mate despite no read quality drop being involved in the read simulation. Longer positional shifts remained limited in magnitude, as reflected by the rapid saturation of the conditional CDF before 20 bp for the forward mate and 40 bp for the reverse mate. Nonconditional error distributions and detailed positional shift distributions are also provided in Suppl. File S1, Section 12 Fig. S11.

### Characterization of structural variation in the DAM gene locus within the pangenome

**Figure 5:**
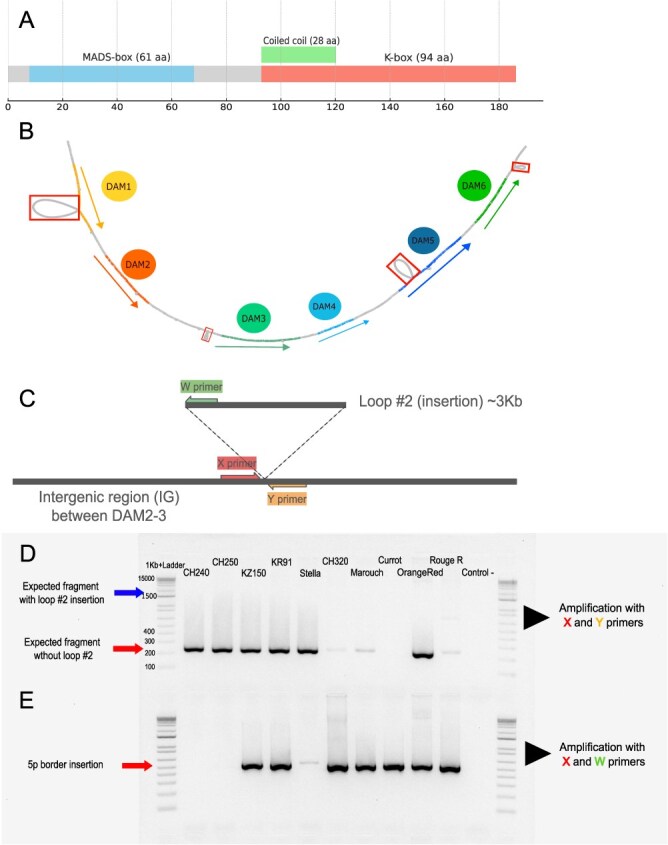
Identification and validation of structural variants over the DAM region. **A.** Schematic representation of a DAM-like gene, here DAM5 in *P. armeniaca* [20]. *DAM* genes show a conserved structure that includes at least a MADS-box (in blue) and a K-box (in red). **B.** The DAM locus was extracted from the graph, with selected nodes ranging from −3000-bp upstream of DAM1 and + 7000-bp downstream of DAM6 (coordinates relative to the Rojo_HCUR path). This zoom-out view on the region highlights with red squares the graph loops that correspond to large structural variations. Nodes corresponding to individual DAM genes are colored and labeled with their corresponding name. **C.** Schematic representation of the amplification approach used to experimentally confirm the loop between *DAM2* and *DAM3.* Arrows indicate the primers flanking the insertion sites and used in PCR. **D and E.** PCR validation of loop #2 between *DAM2* and *DAM3* using the primer pairs X–Y and X–W. **D.** The X–Y pair generates a 240-bp-long fragment when the loop is absent in one (i.e., KZ150 or KR91) or the two haplotypes (e.g., CH240 and CH250). The expected ~3 kb X–Y fragment with loop #2 insertion could not be successfully amplified by PCR because the difficulty in identifying suitably conserved primer binding sites close to the insertion precluded the design of a shorter amplicon. **E.** The X–W pair yields a 541-bp product specific to loop #2 5 prime insertion site, thus demonstrating the presence of loop #2 in one of the KZ150 haplotypes, for example. Conversely, CH240, CH250, and Stella lack the X–W fragment, which indicates the absence of loop #2 in those genomes.

As a showcase of the pangenome utility, we focused on the Dormancy-Associated MADS box (DAM) gene locus to exemplify SVs within the pangenome graph, given their conserved synteny and phenotypic impact [20, 21]. Figure 5A displays the genomic organization of this gene cluster. Despite general conservation, four prominent graph loops, indicative of large indels, were identified over the DAM region: loop #1, a 12-kb insertion within the *DAM 1* gene, was specific to two assemblies CH_240 H2 and RRxCH240_plA H1; loop #2, a 2.1-kb variation in the intergenic region between *DAM 2* and *DAM 3*, was shared across 14 assemblies; loop #3, a 7.1-kb insertion within *DAM 5*, was unique to Rojo_HORA. The fourth loop, downstream of *DAM 6*, was shared among seven assemblies (see Suppl. File S1, Section 13, for details related to all loops). While existing *Prunus* genome annotations provided no specific information for these regions (GDR, www.rosaceae.org), our analysis successfully identified TEs in these indels. Loop #1 contained a high-confidence LTR element (Gypsy-like transposon, 91.53% identity over 886 bp), and loop #3 harbored a TIR element (CACTA-like transposon, 94.12% identity over 170 bp). Conversely, no confident TE matches were found in loops #2 and #4, potentially suggesting different genomic features or highly divergent elements.

To empirically validate our structural annotation, we targeted the loop #2 insertion of the DAM region through genomic DNA amplification using specific oligonucleotide primers flanking the insertion sites (Fig. 5C)**.** We confirmed the presence of the loop #2 insertion in at least one of the two haplotypes of all genomes tested except for CH240, CH250, and Stella, in which loop #2 is absent (Fig. 5D and E). Concerning the artifacts present on CH320, Marouch, and RougeR for the X-Y amplification, genomic analysis, even with a relaxed identity threshold, failed to identify any pair of primer-binding sites that could theoretically generate the ∼200 bp amplicon, even in the genomes showing the faint band. Therefore, the weak band is not supported by a detectable genomic template and is indicative of a PCR artifact, such as a low-efficiency primer–dimer structure, rather than a genuine off-target amplification product.

A notable observation was the complete absence of the DAM gene cluster in the CH_264 assembly within our graph. However, re-examination of PromethION reads (ENA identifier: ERR4656977) for CH_264 confirmed the presence of a DAM gene cluster, suggesting an assembly error, though its precise localization relative to our pangenome remains uncertain.

Therefore, the characterization of SVs within the DAM gene locus highlights the utility of pangenome graphs in uncovering complex genomic variations, particularly those associated with TE activity, and identifies potential assembly inaccuracies.

## Discussion

In this study, we present a comprehensive pangenome of the Armeniaca section, shedding light on both the genomic contributions of the various genotypes and the structural organization that defines this pangenome.

### Assembly quality and curation challenges

The quality of a pangenome graph is fundamentally linked to the quality and meticulous curation of its constituting assemblies [22]. We encountered several challenges during this process, including issues with chromosome labeling and inversions, which necessitated substantial manual intervention. For instance, the GSYX assembly showed signs of chromosome admixture and potential scaffolding issues, as evidenced by its incomplete core genome, a varying synteny, or missing data within the DAM region. Furthermore, the phylogenetic placement of some assemblies, such as ‘Longwongmao’, deviated from expectations. We used a distance-based reconstruction that scales well with sets of complete genomes, but remains less accurate than a more advanced phylogenetic study (e.g., careful selection of orthologous loci and the use of a probabilistic model). This choice also explains the relatively long terminal branch lengths of the tree; they mirror the many k-mers that are not shared by genomes and increase dissimilarity. The inclusion of both phased and unphased genomes in this analysis also warrants caution for downstream genotyping applications, given their potential impact on alignment accuracy and variant interpretation. These observations underscore the critical need for rigorous data curation in pangenomics, as inaccuracies can propagate through analyses and complicate the distinction between genuine biological variants and methodological artifacts.

A similar need for extensive manual curation has been observed in other plant pangenomes, particularly in assemblies rich in repeats or with complex SVs. In *Oryza sativa*, for instance, substantial manual efforts were required to validate and refine genome structure across accessions [23]. Likewise, in *Pisum sativum*, the resolution of centromeric and repeat-dense regions demanded iterative polishing and advanced scaffolding strategies [24, 25]. Alternatively, masking these regions may be another strategy, considering the lack of tools for properly assembling them. These cases illustrate the widespread nature of such challenges in plant pangenomics, particularly in the context of building graph-based references. Meanwhile, these regions must be effectively masked until molecular tools for their efficient assembly become available.

Currently, there is a recognized lack of specialized tools beyond basic read mapping and VCF generation that allow comprehensive quality assessment of pangenome graphs and effective discrimination between true biological variation and technical noise. While this study, along with the associated scripts provided on GitHub, offers some practical guidance, more generalized and scalable solutions remain an unmet need in the field.

### Expanding the Armeniaca pangenome

The Armeniaca pangenome continues to expand with the integration of new genotypes, and we anticipate further growth with the inclusion of additional groups and assemblies. As expected, the incremental integration of diverse genotypes systematically reduced the core genome and expanded accessory regions, consistent with previous reports in plant pangenomics [26, 27]. The observed plateau in cumulative growth following the integration of European (EU) genotypes suggests a saturation of genomic novelty within this phylogeographic group, confirming the diminishing returns of extensively sampling closely related individuals [28]. In contrast, the significant genomic gains derived from the incorporation of Wild (W), Chinese (CH), and particularly Manchurian (Man) genotypes underscore the importance of including genetically distant outgroups to capture broader genetic diversity [11]. A similar pattern was observed in glycine max, where the inclusion of wild relatives (*Glycine soja*) significantly expanded the pangenome and introduced SVs absent from domesticated accessions [29].

This expansion notably occurs at the expense of the core genome, which reached a relatively stable threshold of approximately 27.6 Mb. A similar core genome stabilization has been reported in soybean, where Ref. [29] found that after sampling 27 soybean genomes, the core gene set remained consistent while accessory content continued to increase. The observed stabilization of the core genome suggests the presence of conserved genomic regions that are functionally essential and maintained across all haplotypes of the Armeniaca section. This stabilization trend is consistent with observations in *O. sativa*, where analysis of over 3000 accessions revealed that the core genome plateaued despite the continuous accumulation of accessory variation, indicating a conserved set of essential genes across the species [30]. Importantly, the absence of a definitive logarithmic plateau in cumulative growth across chromosomes further reinforces the observation that continuous pangenomic expansion is likely to happen with the addition of further assemblies, highlighting substantial unexplored genomic diversity.

This expansion was not uniform across chromosomes. When normalized by chromosome length (Suppl. File S1, Section 6), chromosome 5, despite being the smallest, showed the highest density of both conserved and divergent regions, indicating hotspots of structural and sequence diversity. In contrast, chromosome 2 displayed the lowest number of such regions, suggesting a more homogeneous distribution of variation along its length. These differences in chromosomal contribution highlight the importance of considering genome architecture in pangenome analyses and suggest that some chromosomes may play a more pivotal role in shaping intraspecific diversity.

The observed increase in the number of predicted gene models, particularly those derived from *de novo* prediction, within the Chinese (CH) and Sibirica (Sib) phylogenetic groups necessitates careful interpretation. In the CH group, this genomic expansion was inconsistent, being evident in only two of the three assemblies, including the GSYX assembly, whose overall assembly quality remains questionable. In contrast, all assemblies within the Sib group were evaluated as high quality, and the modest gene number expansion appears to be a consistent characteristic across this entire group. This consistent pattern may suggest the presence of novel gene families that are currently under-represented in genomic or transcriptomic studies focusing on the Armeniaca section. However, this hypothesis requires rigorous validation using additional transcriptomic and comparative genomic evidence. Notably, lineage-specific expansions of gene families have previously been reported in individual Armeniaca genomes, including *P. armeniaca* [31], as well as targeted expansions of specific functional gene families in related species such as *P. mume* [32]. These precedents indicate that differential gene family expansion can occur within this clade, supporting the biological plausibility of the observed signal.

The relatively modest genomic contribution of hybrid haplotypes (RRxCH240A and RRxCH240B) supports the understanding that hybrid genomes primarily represent recombinations of parental genomic variation, rather than significant sources of novel genomic content. This observation aligns with gene-centered pangenome analyses in crops such as sunflower, where hybridization was shown to alter gene presence/absence without dramatically expanding total gene content [33]. In our case, the limited contribution of hybrid assemblies at the whole-genome level—including intergenic and structural regions—indicates that such genotypes can reasonably be excluded from graph construction, as soon as the parental varieties are included in the graph. Additionally, their low fraction of private sequence argues against major sequencing or graph-related artifacts in their representation.

### Chromosome-level pangenome dynamics and variation drivers

The considerable influence of the most genetically distant groups on the volume of identified SNPs and Indels is consistent with previously observed correlations between genetic divergence and geographic or taxonomic distances [34]. The contribution of different assemblies and groups to the pangenome varies significantly across chromosomes. The observation that some chromosomes exhibit a core genome proportion of 50%, while others drop to as low as 11%, coupled with the variable quantity of insertions and deletions across chromosomes (as illustrated in Fig. 2D), warrants further investigation into chromosome-specific evolutionary dynamics in *Prunus*. Interestingly, the CH320 assembly displayed a unique pattern of core/accessory/private proportions across chromosomes, deviating from other members of the Wild group. This suggests a possible ancient hybridization and introgression from *P. sibirica*, aligning with previous hypotheses [8]. This variability could be linked to differential selective constraints, recombination rates, chromosomal rearrangements, or historical domestication and other selective events [8, 35]. Indeed, using SNPs [8] showed that approximately half of the top 0.5% of genomic regions identified as being under selection during domestication map to chromosome 4. Correspondingly, our analysis reveals that the European (EU) group possesses fewer insertions (relative to the Rojo_HCUR reference) on chromosome 4 compared to other groups (Fig. 2D), suggesting that this selective constraint also impacts structural variants. Conversely, an opposite dynamic is evident in chromosome 8 (and to a lower degree, chromosome 5), where non-EU groups exhibit high amounts of supplementary bases (Fig. 2D). This observation regarding chromosome 8 is particularly relevant given that the ancestor of the Armeniaca section (and in general, to the *Prunus* genus) is known to have possessed nine chromosomes (see the inferred ancestral Rosaceae karyotype ARK depicted in [8], with chromosome 8 remaining unstable within this genus since its emergence [36, 37]. The structural variation dynamic observed in our pangenome for chromosome 8 thus confirms this long-term evolutionary trend.

Regarding indel lengths, the strongly conserved distribution patterns around 200–400 bp and 800–1800 bp are particularly intriguing (Suppl. File S1, Section 8, Fig. S6). Rather than reflecting direct selective constraints, these recurrent sizes more likely stem from inherent mutational biases linked to the activity of specific TE families. Such patterns are consistent with the replication behavior and structural features of TEs, as discussed in Refs. [38, 39], which highlighted their pivotal role in genome remodeling through bursts of insertions of specific lengths. Our TE enrichment analysis indeed demonstrated a significant representation of specific TE orders, notably TIRs and LTRs, within these size bins, emphasizing their potential role in shaping genomic diversity via structural rearrangements [40, 41]. The presence of TE fragments, particularly gypsy-like and CACTA-like elements, within SVs surrounding the DAM gene cluster suggests a potential mutational contribution of these elements to local genome rearrangements. While such variants could hypothetically influence phenotypic traits such as dormancy [20, 21], further functional validation is required to assess any causal relationship. The DAM region also served as a practical illustration of assembly completeness evaluation, as demonstrated by the absence of annotation in the CH_264 assembly in this specific graph region.

### Associations between TEs and structural variation

TEs contribute directly to structural variation and gene regulation in plants [42], and the patterns observed in our pangenome are fully consistent with this dual role. DNA transposons, particularly MITEs, are known to undergo recurrent bursts of amplification through gap-repair–mediated replication and trans-activation by distantly related autonomous elements [43], providing a natural source of short insertions such as those prevalent in our SV dataset. TE activity also influences the regulatory landscape as the proximity of TEs to genes can lead to reduced or altered gene expression via TE-associated epigenetic silencing mechanisms or disruption of promoter regions [44]. Furthermore, lineage-specific expansions of TEs are known to modify chromatin composition and accessibility patterns, which are fundamental to regulatory divergence between lineages [45]. In *Prunus*, TE insertions have been shown to drive phenotypic divergence, including sweet kernel taste trait-associated MITE insertions in almond [46], highlighting their functional relevance within the *Prunus* genus. Our findings extend this view by demonstrating that SV-associated TEs preferentially accumulate in gene-proximal regions (Fig. 3C) and maintain highly stable family profiles across haplotypes (Fig. 3D). This indicates that TE-mediated structural variation preferentially engages regulatory regions, thereby maximizing its potential impact on gene expression. While Ref. [47] demonstrated distinct TE dynamics (in particular, for LTR-RT) across nine Rosaceae species (including *P. persica* as a representative of the *Prunus* genus), our study focuses specifically on the Armeniaca section and confirms the role of SV-embedded TEs in driving genome-wide evolution through gene regulation.

### Pangenome mapping advantages and limitations

Pangenome graphs offer a clear advantage over single-reference analyses for SNP and SV detection, and significantly improve read mapping efficiency. Our findings indicate that mapping to the graph increased the number of mapped reads by 0.5% to 9.2%, depending on read depth. However, it is crucial to acknowledge that conventional metrics for evaluating mapping quality, such as MAPQ, are software dependent and not directly comparable between graph-based and linear reference-based approaches. This increase in mapped reads can enrich downstream variant calling and associative analyses based on SVs from the graph, as demonstrated in other species like cucumber, barley, and vine [14, 34, 48]. Overall, these results confirm that graph-based representations are more capable at capturing large insertions or deletions that can be missed by linear-reference pipelines, and confirm similar observations made in tomato [49] or in maize [50] pangenomes. Beyond mapping gains, our evaluation of positional accuracy of the mapping reveals that even for the more divergent Sib group, graph mapping maintains a strong coordinate precision below the 50-bp threshold that generally defines SV (Fig. 4B). Unexpectedly, we uncover a drop in precision in the second mate of read pairs. Notably, our read simulations with the VG tool did not involve quality simulations (e.g., Phred scores), and both mates were expected to be processed in the same manner. It is currently unknown if this bias may be related to some algorithmic or implementation choices made in the mapping tool. Despite this observation, overall mapping quality is high, and all tested haplotypes showed more than 97% of remapped reads with more than 95% of positional shift below 10 bp (Suppl. File S1, Section 12). This supports the view that pangenome graphs allow accurate mapping, even for short reads, as long as they integrate a balanced phylogenetic representativeness of the targeted clade. These advantages come nonetheless with substantial supplementary CPU costs (Suppl. File S1, Section 14; Fig. S12), a point on which graph-based methods should improve.

Interestingly, we observed diminishing mapping gains for samples based on higher-depth sequencing—but still gains, though. While this appears counterintuitive at first, the general sampling principles behind genome skimming approaches [51] may be an explanation for this observation. Briefly, genome skimming is used when sequencing capacity per sample is low (few reads) and the information held by genomic elements with high copy numbers are sufficient for the targeted question (taxonomy or populational signals). The repetitiveness of these elements in the genome tends to increase their chance to be randomly sampled in a nontargeted approach, and this sampling bias is consolidated when an assembly strategy is used [51]. As a consequence, low depth sequencing tends to extract a large proportion of repeated elements such as organelles, nuclear rRNA tandem repeats, or different families of homologous repeats such as TEs. By making the hypothesis that our low coverage samples follow this trend (the large proportions of TEs that we identified from our sample tend to support this hypothesis), it is expected that a larger diversity of reads could be mapped to the graph and not the reference genome, as the graph should better represent the diversity of apricot repeats. These observations also raise a warning for posterior association studies: they could be biased toward certain genomic elements, such as satellites and transposons in the samples of lowest depths [52].

## Conclusion

Our comprehensive analysis underscores the critical importance of broad genetic sampling and meticulous structural characterization for a complete understanding of genomic diversity and evolution in general and in the Armeniaca section in particular. Future studies integrating additional diverse genotypes, particularly through careful selection of accessions aiming at filling identified diversity gaps, and incorporating phenotyping for associative analysis, will greatly benefit from the construction of this super-pangenome. Graph-based SV datasets have demonstrated strong utility for GWAS in crops, including barley [34], highlighting their potential for unlocking previously unexplored genotype–phenotype associations missed by standard SNP-based analysis. The dataset built in this study will enable us to fully leverage the observed genetic diversity for apricot crop improvement. Specifically, the interplay between SVs and TEs is crucial, as TE mobilization and insertion drive many SVs, often leading to the rearrangement of regulatory elements that ultimately modulate gene expression and contribute to phenotypic variation. Therefore, this work not only provides an unparalleled resource for the Armeniaca section but also confirms the necessity of high-resolution, graph-based pangenomics to fully capture and leverage the structural genetic diversity required for effective crop improvement strategies.

## Materials and methods

### Genomic dataset

In total, long-read assemblies for 30 Armeniaca genomes were compiled: 15 of *P. armeniaca*, 5 of *P. sibirica*, 1 of *P. mandshurica*, 1 of *P. hongpingensis*, and 1 of *P. zhenghensis* (Suppl. Table T1). Out of these, 25 were included into the pangenome graph (Fig. 1). For this study, we *de novo* assembled seven accessions: two wild Central Asian *P. armeniaca* (KZ_150_8 and KR_091_1 from W2 and W1 genetic clusters, respectively [8]), two *P. sibirica* genomes (CH240 and CH250), and two hybrids (Table 1). All data sources and sequencing approaches for the 32 assemblies are detailed in Suppl. Table T1. Detailed methods for the new assemblies can be found in Suppl. File S1, Section 1.

### Assemblies curation

For each chromosome of each genome assembly, we retained the longest scaffolds, for a total of eight chromosome sequences per genome. Prior to the pangenome construction, we verified the integrity of each sequence using two different steps. First, we clustered all sequences using their similarity and via the method implemented in the PGGB sequence partitioning script [53]. Briefly, we compressed and indexed our files using samtools v1.10 [54], fastix v0.1.0 (https://github.com/ekg/fastix), and used mash v2.2.2 [55] to obtain a distance matrix, using the option ‘-s 10000’ to specify the sketch size (the amount of representative k-mers summarizing each genome). We then inspected the k-mer distance matrix to evaluate the similarity between all assemblies and chromosomes, as a means to confirm the correctness of their labeling. Distance heatmaps and dendrograms were generated using R [56] and the package pheatmap v1.0.12. Where needed, we renamed the mislabeled chromosomes following the standard nomenclature for *P. armeniaca* genetic map (Genome Database for Rosaceae, https://www.rosaceae.org) and we harmonized chromosome orientations with seqtk v1.3 (https://github.com/lh3/seqtk) using the -r option. We verified global synteny between chromosomes with the D-genies v1.5.0 tool [57], using the web version (https://dgenies.toulouse.inra.fr). This was complemented by differentiation of inversion, duplication, and translocation events using SyRI (v.1.6.3) [58] and the resulting alignments visualized using Plotsr (v.1.1.1) [59].

### Assemblies annotation

The genomic assemblies selected for pangenome graph construction were annotated using the NCBI’s EGAPx pipeline (available on https://github.com/ncbi/egapx). Evidence utilized for constructing the gene models was aggregated from two primary sources. First, all previously documented gene models within the entire *Prunus* genus were incorporated by specifying the corresponding NCBI taxonomic identifier within the EGAPX configuration (taxid: 3754). Second, 15 RNA-seq experiments (NCBI Bioproject: PRJEB42479) were included. These datasets corresponded to transcriptomes derived from various tissues and developmental stages in *P. armeniaca,* supplemented by two transcriptomes from *P. mandshurica* and *P. sibirica*. Consistent with the EGAPX recommendations for performing annotation across multiple species, the criteria for RNA-seq dataset alignment were relaxed as follows: rnaseq_collapse: -high-identity 0.8; sam2asn: -filter ‘pct_identity_gap > = 85’; star_wnode: -pct-identity 85.

### Strategy for iterative chromosome graph construction

We build an independent pangenome graph for each chromosome using Minigraph-Cactus [60]. This tool relies on an iterative approach in which a guide tree defines the order of graph augmentations, each assembly being iteratively aligned and introduced to the graph. By design, each ordering can potentially end in a different final graph (see [61]). As a consequence, two criteria will impact the graph construction process: (i) the choice of the ‘reference’ (starting) assembly, recommended being of high quality and/or richly annotated, and (ii) the order of assemblies for graph augmentation, which should ideally represent phylogenetic proximities, so that potential genome alignment artifacts may not be introduced.

To define the order of following assemblies, we followed their degree of genetic divergence relative to Rojo_HCUR, our selected reference assembly and the starting point for pangenome graph augmentation. To do so, we computed k-mer distances between assemblies using the PanTools method [62]. Briefly, for each chromosome, the KMC [63] and the PanTools ‘k-mer classification’ commands were used to obtain a Mash distance matrix between all chromosomes (k-mer_size = 19). These distances were used to build a phylogenetic reconstruction that mimics the approach used by mini-graph-cactus to determine assembly alignment order but allowed us to carefully investigate the resulting tree for potential unexpected placements. The corresponding neighbor-joining phylogenetic tree was built with the phangorn v2.11.1 [64] and ape v5.1-7 R packages [65].

Each chromosome tree was visually inspected for inconsistencies with Dendroscope v3.8.10 [66], while the Robinson-Foulds (RF) distance [67] was used to verify topological consistency between trees. The online tool iTol [68] was used to generate tree figures, and taxonomic relationships were compared with the Rehder taxonomic classification of Armeniaca [69]. This also helped to approximate taxonomic relationships for several assemblies that were not listed by this source.

### Pangenome graph construction

We used the singularity image v3.9.9 [70], retrieved from https://github.com/sylabs/singularity, that included the Minigraph-Cactus pipeline v2.6.13. For each run per chromosome, the NJ phylogeny (with outgroups pruned) was given as input to the pipeline, ensuring controlled ordering during construction, and Rojo_HCUR was set as the starting assembly. Two important parameters were manually set: filter 1 allowed all nodes to be retained in the graph, including those that are unique to a single haplotype, and reference was set to a list containing all haplotypes in their phylogenetic order, ensuring that all bases are represented in the final graph. This is crucial for population-level analyses, where genetic diversity across individuals needs to be retained. Other noticeable options used are all-snarls to include all complex variations in indexes that will be associated with the graph and gbz-translation to pack the graph in gbz format, where all haplotypes can be translated into our reference assembly coordinates. Finally, Minigraph-Cactus produces three output graphs by default (‘full’, ‘clip’, and ‘filter’, see tool documentation). Every result from our study is based on the ‘full’ graph, where all bases from input assemblies can be retained.

Simultaneously with its construction, VCF (Variant Calling Format) files were generated. Because a VCF will project graph variants to sequence coordinates of a single selected assembly, we produced as many VCFs as assemblies in the graph. While one could directly call the options vcf and vcfReference to do so, we experienced issues with the singularity image (the vg deconstruct command was failing in some cases). Consequently, we ran these extractions manually after construction, using a more recent version of the vg toolkit (compared to the version included in the Minigraph-Cactus singularity image) [71]. First, we extracted the paths with command ‘vg paths’ to obtain the normalized names of all graph paths (e.g., normalized assembly names). Then, we used vg v1.57 to perform the ‘vg deconstruct’ command and obtain the targeted VCFs.

### Graph statistics and variants extractions

Graph and variant statistics were generated with four approaches. First, we used the Pan1c workflow (https://forge.inrae.fr/genotoul-bioinfo/Pan1c/pan1c) using all chromosome graphs as inputs. This workflow contains a module to generate general and pangenome-oriented graph statistics, as well as provide visualization tools to estimate the quality of the graph. Second, we ran the Pansel tool [72], which aims to extract graph regions holding significantly conserved (conversely diverging) regions, relatively to the full content of a graph. We ran the tool using Rojo_HCUR as the reference and used the default window size of 1000. As in the original paper of the method, only the extreme 5% of the fitted log-normal distribution was retained to define conserved and divergent regions. We ran the method once per chromosome graph (to detect conserved/divergent regions within) and once for the full pangenome (to detect these regions among chromosomes). Third, we ran vcftools v0.1.16 [73] on the VCFs files built from the projection to the ROJO_HCUR assembly. We obtained insertions/deletions (indels) statistics with the option --keep-only-indels and the SNP statistics with the option --remove-indels. Results were analyzed using the R dplyr [74] and ggplot2 [75] packages. We analyzed indels within a size bin of 50 to 100 000 bp, this upper bound being the maximum length that the pan1c workflow will consider, thus allowing direct comparison between the tools. Finally, Panacus plots [76] were generated to estimate the variant enrichments brought to the graph by each assembly. Assembly order was set manually using the -O option and followed the ordering used at construction. The -q parameter was used to define quorum thresholds, which correspond to the minimum proportion of haplotypes in which a genomic segment must be present to be included in the output. A quorum of 1.0 (i.e., 100%) identifies the core genome, comprising regions shared by all haplotypes, while a quorum of 0.0 includes any sequence present in at least one haplotype, thus representing the entire cumulative amount of new bases brought by each haplotype.

### Transposon content

To identify potential transposons, the extracted indels were binned by size, and each subset was then independently analyzed using BLAST version 2.2.28 [77]. This analysis was complemented by a custom transposon library built from 11 assemblies selected to maximize genetic divergence. Briefly, for each of these assemblies, we used the TEdenovo pipeline [78] from the REPET package v3.0 (https://urgi.versailles.inrae.fr/Tools/REPET) to generate a new transposon library, followed by three rounds of curation based on TEannot [79, 80], where any consensus not supported by at least one full-length copy in the assembly was discarded [81]. The 11 curated libraries were then concatenated into a pan-library of apricot species, and a final redundancy filtering step was performed using PASTEC [82] to retain a single representative for each transposon definition sharing ≥98% of length and ≥ 95% of sequence identity. Using this custom library, we used BLAST version 2.2.28 [77] to obtain the best transposon alignments for each indel. To select the most robust and relevant transposons for each indel, we applied a set of hierarchical criteria designed to minimize redundancy and enhance result interpretability. First, only transposon hits with a minimum sequence identity of 90% were retained to ensure high-confidence matches. Then, priority was given to transposon size (larger elements being more likely to represent complete and informative annotations), followed by lower e-values, higher sequence identity, and longer alignment lengths. In cases of equal ranking, alignments with fewer mismatches and fewer gaps were preferred. Hits without an assigned order or class were also excluded from the dataset to maintain classification consistency across the analysis.

To allow meaningful comparisons of transposon density across both indel size bins and chromosomes, independently of differences in available sequence space, we applied two complementary normalization strategies. First, the number of transposons detected within each indel size bin was normalized by the total aligned base pairs in that bin (align_len), accounting for the fact that larger bins naturally contain more sequence and may therefore accumulate more hits. Second, transposon counts per chromosome were normalized by the average chromosome length, using size data derived from high-quality haplotype assemblies.

These normalization steps enhanced the biological interpretability of the density patterns observed. Variability and confidence intervals of the proportion of transposons within indels and chromosomes were estimated through a bootstrap procedure of 1000 replicates each. To test for statistically significant differences, we performed Pearson’s chi-square tests, which confirmed a significant pattern between TE order distributions and both indel size bin and chromosome. In addition, pairwise proportion tests were carried out for the top 5 TE orders between different bins and chromosomes.

### Investigating structural variation, TEs, and gene proximity

To investigate the relationship between SVs, TEs, and their proximity to coding genes, we contextualized insertions and deletions (50 to 100 000 bp) relative to the Rojo_HCUR reference genome using TE annotations generated by the REPET v3.0 tool (https://urgi.versailles.inrae.fr/Tools/REPET). First, all indels were extracted from the pangenome graph using the vg deconstruct method [71], before being filtered to retain only SV ≥50 bp using VCFtools v0.1.16 [73]. In parallel, TE annotations were independently generated on the linear sequences of one representative haplotype per distinct genetic groups: Pman (for the Mandshurica group), KZ150 H1 (for the W group), and CH250 H1 (for the Sib group). This annotation employed a curated REPET library. The distances between the annotated TEs and their nearest non-TE coding genes were calculated, and positional relationships were categorized as upstream, downstream, and overlap as detailed in Ref. [18].

To integrate gene annotation, TE annotations, and SV extracted from the pangenome graph, custom scripts and the ‘vg find’ command were used to establish a genome coordinate relationship between the SVs extracted from the graph and the coordinates on the individual haplotypes. These haplotype coordinates were then compared with the annotated TE coordinates to identify SV-embedded transposons and determine their distance to the nearest non-TE gene. For example, an insertion in KZ150 H1 relative to Rojo_HCUR corresponds to one position in the Rojo_HCUR genome but two positions (the beginning and end of the insertion interval) in KZ150 H1. If this interval contains a TE annotated by REPET, the proximity of that TE to the nearest gene can be calculated. By iterating this process for all SVs, a distribution was generated that reflects the proximity between the SV-embedded transposons and coding genes. We began with a general description of the TE families positioned downstream and upstream of the genes, then the focus was placed on TEs categorized as ‘upstream’ or ‘upstream & overlap’ to evaluate potential regulatory impacts on gene expression.

Analyses were performed in R v4.3.2 using data.table [83]. TE annotations and haplotype-specific SV intervals were intersected with ≥10-bp overlap. SV coordinates were taken directly from *vg find* and corrected with GFA node lengths to ensure accurate linear projections. TE families were grouped into five biological orders (LTR, TIR, LARD, TIR|LTR, Unclassified). Only the 12 most abundant TE families were individually colored, with all others collapsed as ‘Other’.

### Short-read mappings

To evaluate the potential of our pangenome graph in thoroughly detecting SVs, we compared the mapping of a panel of 322 Illumina short-read accessions from Ref. [8] to the graph vs the reference assembly Rojo_HCUR. This panel represents the diversity of the Armeniaca collection maintained at the French CRB at Bordeaux and was sequenced at depths of up to 20×. To allow reads to map preferentially to the chromosome holding the most similar sequence, in particular when homologous sequences (or translocations) are present on more than one chromosome, all chromosomes were combined. We concatenated all Rojo_HCUR chromosomes into a single FASTA, subsequently indexed with samtools faidx and minimap2 -d. Read mapping on the reference was performed using minimap2 v2.28 [84]. Mappings were computed using the short-read parameter preset (-ax sr) and the parameter (-R) keeping read group information for downstream processing. Sample metadata were introduced in the resulting BAM files using samtools view -bh, ensuring compatibility with following analyses. The mapping to the graph was performed using the VG toolkit v1.20 [85]. Before mapping, chromosome graphs were combined using vg combine -p. The combined graph was then indexed with vg autoindex and the giraffe pipeline (-w giraffe) to generate a GBZ binary. The read mapping was performed using vg giraffe with paired inputs (−p) and output alignments in GAM format. We also set the -N and -R options to assign sample identifiers to the results. To allow direct comparison between the two approaches, each GAM was then projected to Rojo_HCUR sequence coordinates, by generating a BAM file (e.g., graph-based coordinates are translated into linear coordinates of a selected reference).

We used samtools 1.20 [54] to compute general statistics from the BAM output of both methods. To achieve a fair comparison between Minimap2 and VG Giraffe results, we carefully set up the FLAG-based filtering by applying the ‘-F 2308’ filter to all BAM files (see Suppl. File S1, Section 10, for more details). Mapped read counts and associated MAPQ score distributions were then extracted using ‘samtools view’ and ‘samtools stats’ commands. Sequencing depth from the 322 short-read samples was approximated using ‘samtools coverage’ and via their mapping to the Rojo_HCUR reference.

Positional accuracy was estimated using a strategy inspired by Ref. [19] and based on a leave-one-out approach. Three accessions were selected from three groups of increasing divergence from the reference: Marouch (EU), KZ150 (W), and CH240 (Sib). Using the graph of chromosome 1, short reads were simulated from the corresponding haplotypes (H1 and H2) paths using the ‘vg sim’ command from the VG toolkit (paired-end, 150-bp, 350 ± 50-bp inserts). Resulting reads were annotated via custom scripts with graph node identifier, start position in this node, and equivalent position in the reference Rojo_HCUR when possible. Three ‘depleted’ graphs (EU-dep, W-dep, Sib-dep) were subsequently and independently generated by removing the genome paths from the aforementioned groups. Simulated reads were remapped to depleted graphs using ‘vg giraffe’ and default parameters. Mapping recall was measured using the number of reads producing an alignment above quality 10. Only read pairs for which both mates were successfully mapped were retained for accuracy analyses. To quantify mapping accuracy, the positional error Δ was defined as the difference (in base pairs) between the mapped 5′ reference coordinate of the simulated forward and reverse fragments and their position of origin in the graph, both relative to the reference Rojo_HCUR coordinate system. Absolute positional errors |Δ| were then computed for all reads and derived to an empirical cumulative distribution function (CDF).

### Structural variation over the DAM region in Armeniaca

To extract the tandem-arrayed Dormancy-Associated MADS box (DAM) gene region from the graph, we performed a homology analysis based on DAM1 to DAM6 genes. We used Uniprot sequence identifiers from Ref. [20], with DAM1 to DAM4 and DAM5 to DAM6 originating from *P. mume* and *P. armeniac*a*,* respectively. Candidate homologous regions were identified by aligning gene nucleotide sequence to all assemblies using BLAST+ v2.2.28+ [86] as well as, for DAM5 and DAM6 only, translated proteins of the MADS box domains extracted from UniProt (identifiers: A0A4D6CKB8_PRUAR; A0A4D6CHU2_PRUAR). We checked the validity of these candidate regions by aligning their sequences to known DAM coordinates (i) from the Marouch assembly, as well as (ii) annotated DAM regions for all assemblies referenced in the Genome Database for Rosaceae (GDR). We retained alignments with the highest identity and length to define putative DAM coordinates. As Rojo_HCUR is not available in GDR, we used BLAST coordinates from the Marouch assembly, easier to inspect via JBrowse, to retrieve node IDs for each DAM gene. These were then mapped onto the subgraph previously extracted from Rojo_HCUR coordinates using the VG toolkit. Subgraphs were visualized with Bandage [87].

The gene cluster structure was verified utilizing the JBrowse tool integrated within the Genome Database for Rosaceae (GDR), as described by Jung *et al.* [88]. This platform facilitated the comprehensive visualization of available transcriptomic data, enabling its direct correlation with BLAST outputs. Furthermore, JBrowse provided crucial insights into the architecture of the assemblies, specifically aiding in the identification and characterization of genomic rearrangements (e.g., loops) present in only a subset of the assemblies.

To unequivocally confirm the presence of the DAM region in the *P. mandshurica* CH 264 genome, despite its absence from its primary assembly, we used a targeted approach using PromethION long reads (ENA accession: ERR4656977). The raw ONT sequencing reads were used to build a BLAST database. Reads matching the DAM regions were then identified by searching the database using candidate DAM sequences as queries. The specific commands and methodological details for this analysis are comprehensively documented in https://forge.inrae.fr/benjamin.linard/armeniaca_pangenome_paper.

### PCR verification of the DAM indel

PCR reactions were prepared in a final volume of 20 μl, containing 20 ng of template DNA, 1× reaction buffer, dNTPs at a final concentration of 0.2 mM each, MgCl₂ at 2.5 mM, and forward and reverse primers at 0.2 μM. Jumpstart DNA polymerase (Sigma-Aldrich) was included at 1 unit per reaction. Thermal cycling consisted of an initial denaturation step at 94°C for 5 min, followed by 37 cycles of 30 sec denaturation at 94°C, 1-min annealing at 62°C, and 2-min elongation at 72°C. The primers sequences are Primer X: AGGTTTTGACGGAACCTAAGAG, Y: GTGTTT(C/T)TAAAGTCCATTTCATATGAGG, and W: GATGGGGTTTGGTGGTTTGC.

### Artifact verification

To determine if the faint ~200-bp bands observed in a subset of genotypes were products of genuine off-target amplification, an exhaustive *in silico* search for secondary amplicons was performed using the primer sequences. A BLAST (v2.2.28) nucleotide database was constructed from all available genome assemblies, and the primers were aligned using blastn-short with stringent parameters (≥95% identity, word size = 7). A custom filtering pipeline then evaluated all possible Primer X and Primer Y combinations that occurred on the same contig in opposite orientations (consistent with PCR). The pipeline specifically targeted genomic distances between the primer sites compatible with the artifact’s size (100–1000 bp, focusing on the 150- to 250-bp window) to uncover potential off-target binding sites.

## Supplementary Material

Web_Material_uhag104

## Data Availability

All the relevant data supporting the findings of this study are available in the paper and supplementary files: Suppl. File S1 paper complements and the associated tables and datasets. All scripts and their methodology can be found on https://forge.inrae.fr/benjamin.linard/armeniaca_pangenome_paper; data associated with the building of the graph are available on https://doi.org/10.57745/WUCADU. Short-read sequencing data are detailed in extended Table 1 [8]. All the raw sequencing data generated during the current study (i.e., long-read PACBIO sequences of KZ150, KR091, CH240, CH250, RougeR, RRxCH240 A & B) were deposited at the European Nucleotide Archive (ENA) under accession number PRJEB103076. Their assembled and annotated versions were transferred to the Genome Database of Rosaceae (GDR) for release upon publication. Additional data, not yet deposited in public repositories, can be obtained from the authors upon reasonable request.
