# Supplementary material for: Phylogeny-driven pangenome analysis uncovers the genomic landscape of domesticated and wild Armeniaca species
Source: Hortic Res. 2026 Mar 27;13(7):uhag104. doi: 10.1093/hr/uhag104 (PMC13283847; doi:10.1093/hr/uhag104)
Supplement: Web_Material_uhag104 [file web_material_uhag104.zip › (revised) Suppl_File_S1__paper_complements.docx]

**Supplementary File S1**

Phylogeny-driven pangenome analysis uncovers the genomic landscape of domesticated and wild Armeniaca species

Corresponding authors: veronique.decroocq@inrae.fr ; benjamin.linard@inrae.fr

**Table of contents**

[**Section 1 : De novo genome assemblies for KZ150, CH250 and KR091 2**](#_rzvdrhiwqh8k)

[**Section 2 : Chromosome synteny 4**](#_ijppwfbmwix3)

[**Section 3 : Assemblies corrections 9**](#_cnlhg7k3w4lo)

[**Section 4: Genetic distances relative to reference assembly Rojo_HCUR 15**](#_vbpg1df208kb)

[**Section 5: Phylogenetic tree of selected assemblies 16**](#_aejf6ds3fq1k)

[**Section 6 : Summary of Graph Metrics 18**](#_168zd6mr9vms)

[**Section 7: Complements on P. armeniaca transposon library generation 19**](#_2fq6ntffc866)

[**Section 8 : Supplements on SV statistics 20**](#_yld2xw2ntb0k)

[**Section 9 : Supplements on transposons analyses 22**](#_z7b60citzka9)

[**Section 10 : Filtering of reads after mapping 25**](#_ml40m2feqkbn)

[**Section 11 : MAPQ scores distribution following mapping 28**](#_hh6vyvjrfmo0)

[**Section 12 : Complements on the evaluation of mapping accuracy 29**](#_8haby31fo0f8)

[**Section 13 : Data relative to the DAM Genomic Region 31**](#_86x4b178e6yp)

[**Section 14 : Computational cost of mapping 32**](#_rc1x90810cp3)

#

# Section 1 : De novo genome assemblies for KZ150, CH250 and KR091

High-fidelity (HiFi) long-read sequencing data generated using PacBio technology were employed for the de novo assembly of six Prunus armeniaca (apricot) genomes ( RougeR, KR091, KZ150, CH240, CH250, RRxCH240). For four of these genomes (RougeR, KR091, RRxCH240A and RRxCH240B), assemblies were generated using the Asm4pg v1.1.0 pipeline (<https://forge.inrae.fr/asm4pg/GenomAsm4pg>), which incorporates integrated steps for quality control, assembly, and polishing optimized for plant genomes. Genome assembly was performed using hifiasm (v0.16) [(Cheng et al., 2021)](https://www.zotero.org/google-docs/?fl96JZ), followed by scaffolding with RagTag (v2.0.1) [(Alonge et al., 2022)](https://www.zotero.org/google-docs/?O3Y34k), using the reference genome ‘Marouch#14’ as a guide. Scaffolds were further anchored and ordered using ALLMAPS, based on high-density genetic maps from [(Groppi et al., 2021)](https://www.zotero.org/google-docs/?8OhWEm). This approach enabled the placement of over 90% of assembled sequences onto chromosomes.

For the remaining two genomes (KZ150 and CH250), additional data types were incorporated to enhance assembly quality. Long-range scaffolding was performed using optical maps (790X and 3200X, respectively), while Illumina short reads were used for polishing and error correction [(Groppi et al., 2021)](https://www.zotero.org/google-docs/?Oczbhd). This strategy enabled the anchoring of all sequences onto the sixteen apricot chromosomes of each diploid genome. In summary, for the KZ150 and CH250 genome assemblies, high-fidelity long-read sequencing data (HiFi reads) were assembled using hifiasm v0.15.2 (<https://github.com/chhylp123/hifiasm>) with default parameters to generate two haplotype-resolved assemblies (hap1 and hap2). The resulting GFA-format graphs were converted into FASTA sequences using gfatools v0.4. At this stage, the assemblies consisted of 745 contigs for hap1 and 333 for hap2, with total assembly sizes of 254.2 Mb and 243.8 Mb, respectively. The N50 values were 11.6 Mb (hap1) and 10.3 Mb (hap2). Detailed scaffold statistics were computed directly from the hifiasm output. Hybrid scaffolding, based on the optical maps, was, in a second stage, performed at CNRGV (Centre National de Ressources Génomiques Végétales, INRAE Centre de Occitanie-Toulouse), integrating genetic maps with the initial assemblies. Paired-end Illumina reads were quality-filtered using fastp and mapped to the scaffolded assemblies using BWA-MEM. Average insert size (269 bp) and read length (151 bp) were estimated from Qualimap reports. Gap closing was conducted using GapCloser with a configuration file based on empirical insert size and read parameters. This process significantly reduced the number of ambiguous 'N' bases: from ~7.9 million to ~37,000 in HS1, and from ~1.6 million to ~32,000 in HS2. Minor reductions in total assembly size were observed post-gap closure. In silico PCR was then performed using the UCSC isPCR tool against both haplotype assemblies (HS1 and HS2) using a marker set comprising SNPs and SSRs. Results were filtered and used to anchor genetic markers to the scaffolds. In a third stage of the assemblies, the ALLMAPS pipeline (<https://github.com/tanghaibao/jcvi/wiki/ALLMAPS>) was used to order and orient scaffolds into chromosome-scale pseudomolecules based on the integrated genetic maps. PCR results were parsed to generate BED input files. The final assemblies consisted of eight ordered scaffolds per haplotype (HS1 and HS2), representing the eight chromosomes, with total sizes of 218.5 Mb and 214.1 Mb, respectively. Unscaffolded sequences totaling ~2.8 Mb were retained separately. Alignments against a reference genome (Marouch #14) using D-GENIES revealed an inversion on chromosome 7 in both haplotypes. The corresponding chromosome sequences were extracted, reverse-complemented using EMBOSS revseq, and reintegrated into the respective assemblies. Finally, to correct for potential phasing inconsistencies, chromosome 1 was swapped between the HS1 and HS2 assemblies. Individual chromosome sequences were extracted and merged using custom awk-based scripts and cat commands to regenerate the final genome assemblies for each haplotype.

For all genomes, assembly completeness was evaluated using BUSCO (v5.3.1) [(Manni et al., 2021)](https://www.zotero.org/google-docs/?YK5rxp) with the eudicots_odb10 lineage dataset, with all assemblies achieving completeness scores exceeding 98%. The final assemblies exhibited high contiguity, with N50 values ranging from 21 to 29 Mb and L50 values between 3 and 4. Whole-genome alignments were visualized using D-GENIES [(Cabanettes and Klopp, 2018)](https://www.zotero.org/google-docs/?HVxk1c) to assess structural accuracy and, where necessary, correct chromosome orientations.

#

# Section 2 : Chromosome synteny

Global synteny maps were generated with Syri. The assemblies are ordered vertically (top to bottom) following the established phylogenetic arrangement (Figure 1 of the, main manuscript and section 4 below). Three assemblies - Rojo_HUCR and Rojo_HORA from the EU group and GSYX from the CH group - exhibit a higher number of inversions and translocations across most chromosomes. In the case of the RougeR_H1 and RougeR_H2 assemblies, they display either substantial chromosome length variation or elevated amounts of inversions and translations specifically on chromosomes 2, 3, 5 and 7.

The Table below highlights chromosomes and assemblies for which more events are observed.

**Suppl. Table S1: Chromosomes and assemblies exhibiting elevated inversion and translocation events**

| **Chromosome** | **1** | **2** | **3** | **4** | **5** | **6** | **7** | **8** |
| --- | --- | --- | --- | --- | --- | --- | --- | --- |
| **Rojo_HCUR** | X | X |  | X |  | X | X | X |
| **Rojo_HORA** | X | X |  | X |  | X | X | X |
| **GSYX** | X | X |  | X |  |  | X |  |
| **RougeR_H1** |  |  | X |  | X |  |  | X |
| **RougeR_H2** |  | X | X |  | X |  |  | X |

The figures below are Syri outputs for each chromosome, chromosome 1 being available in the main text.

A.
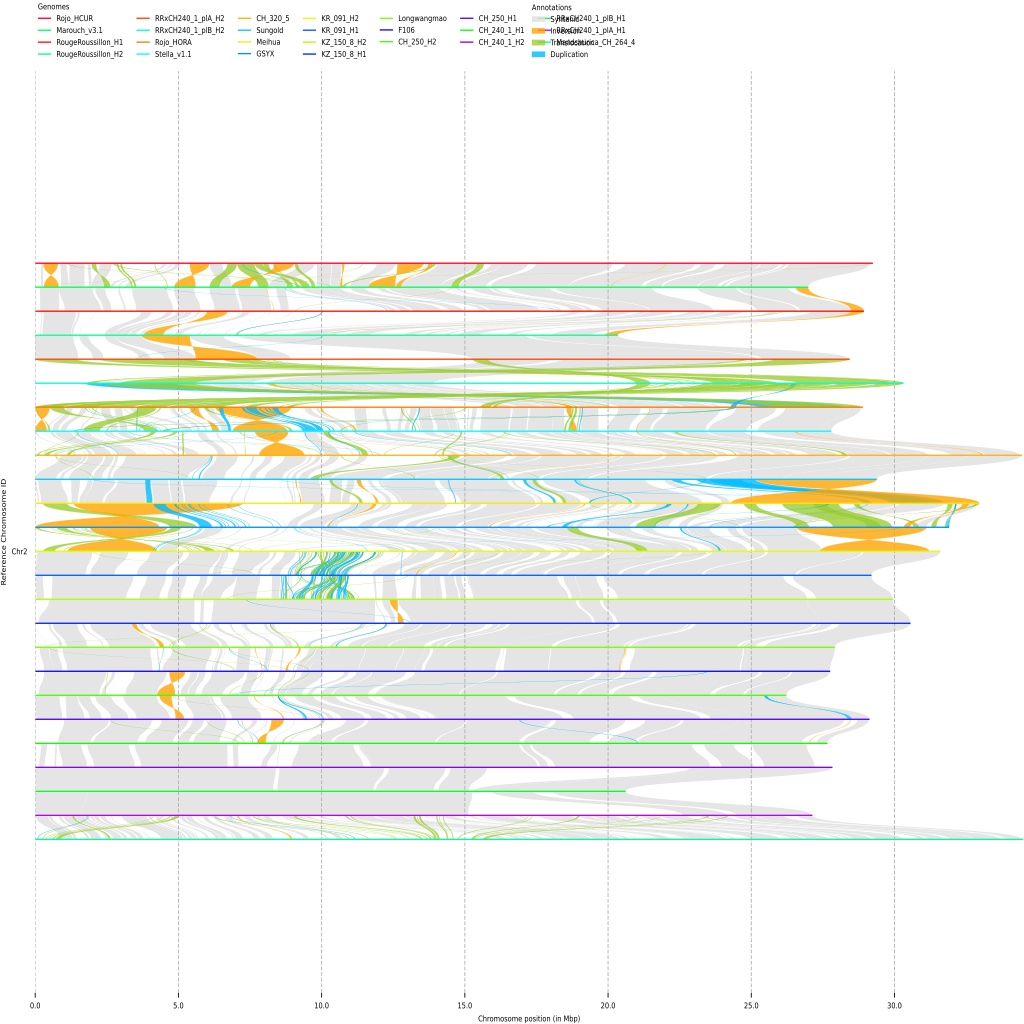


B.
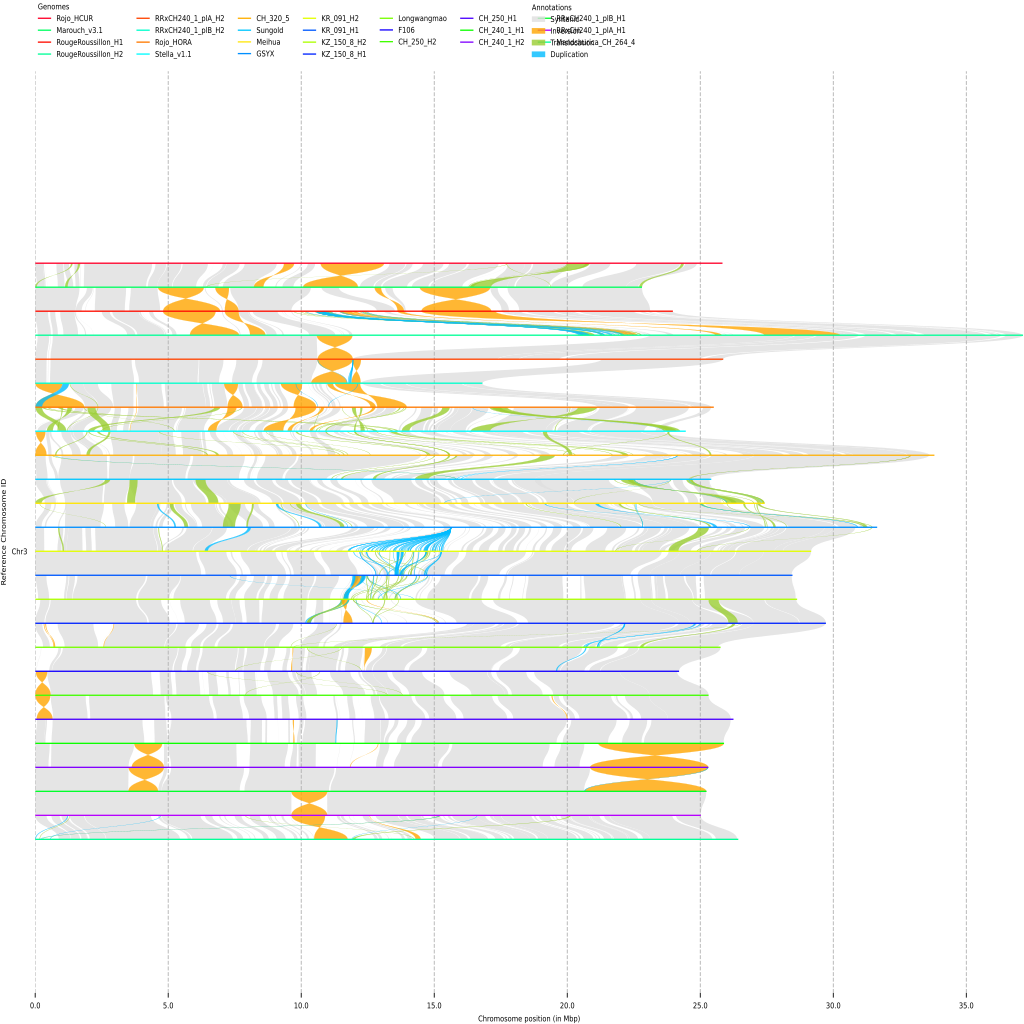


C.
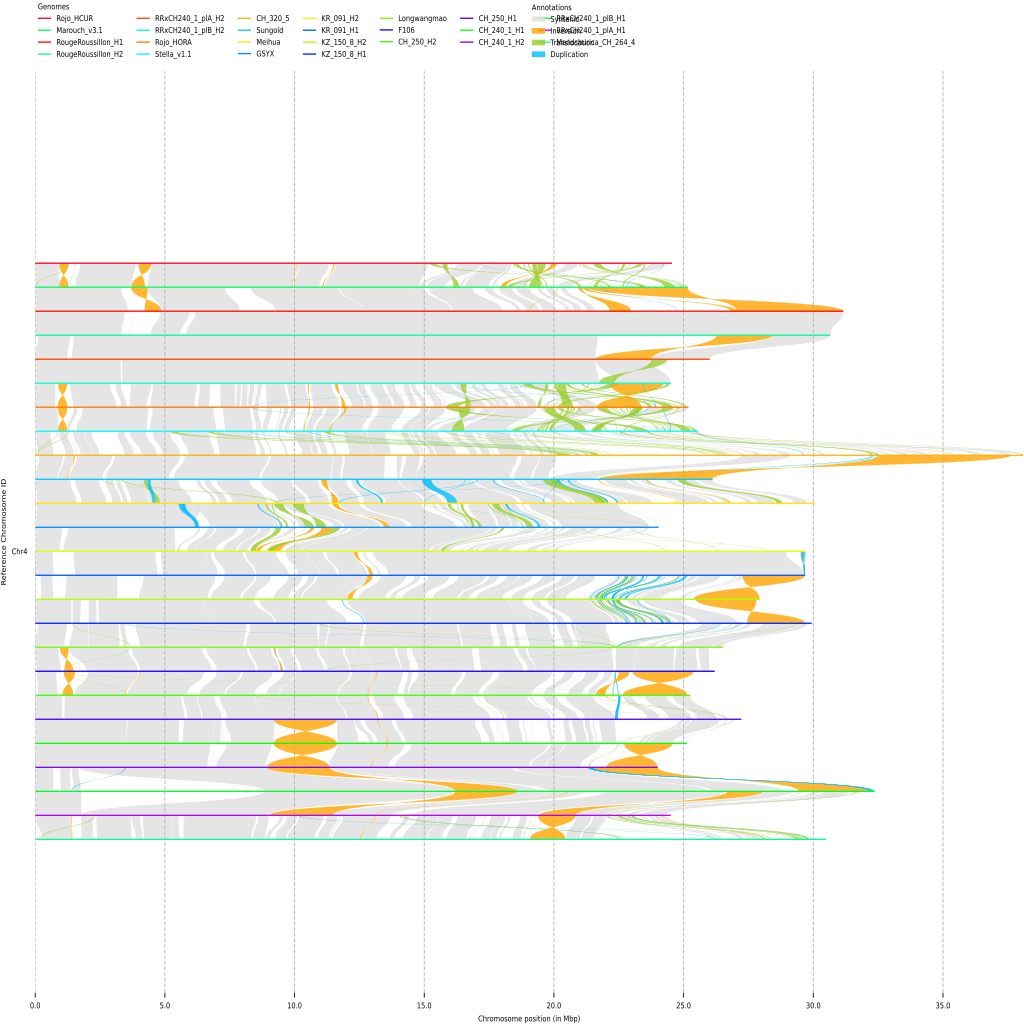


D.
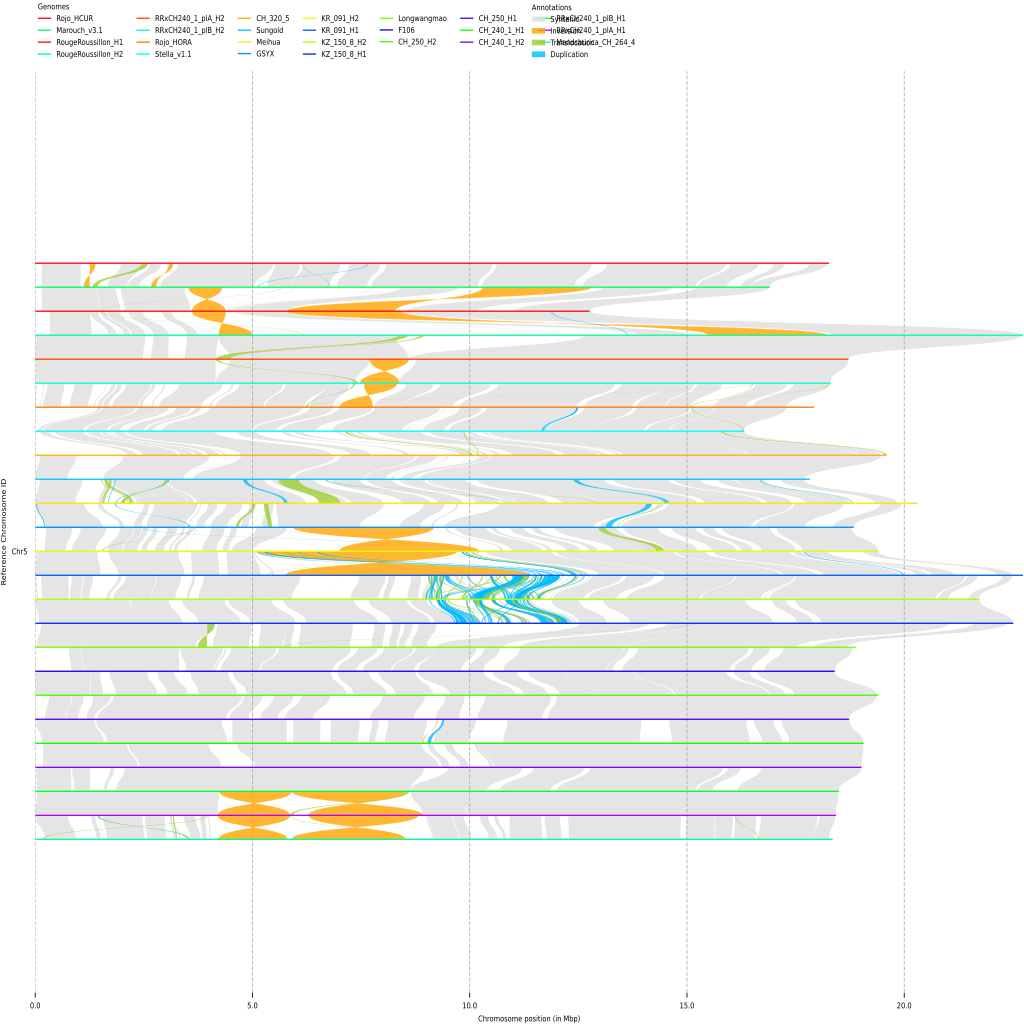


E.
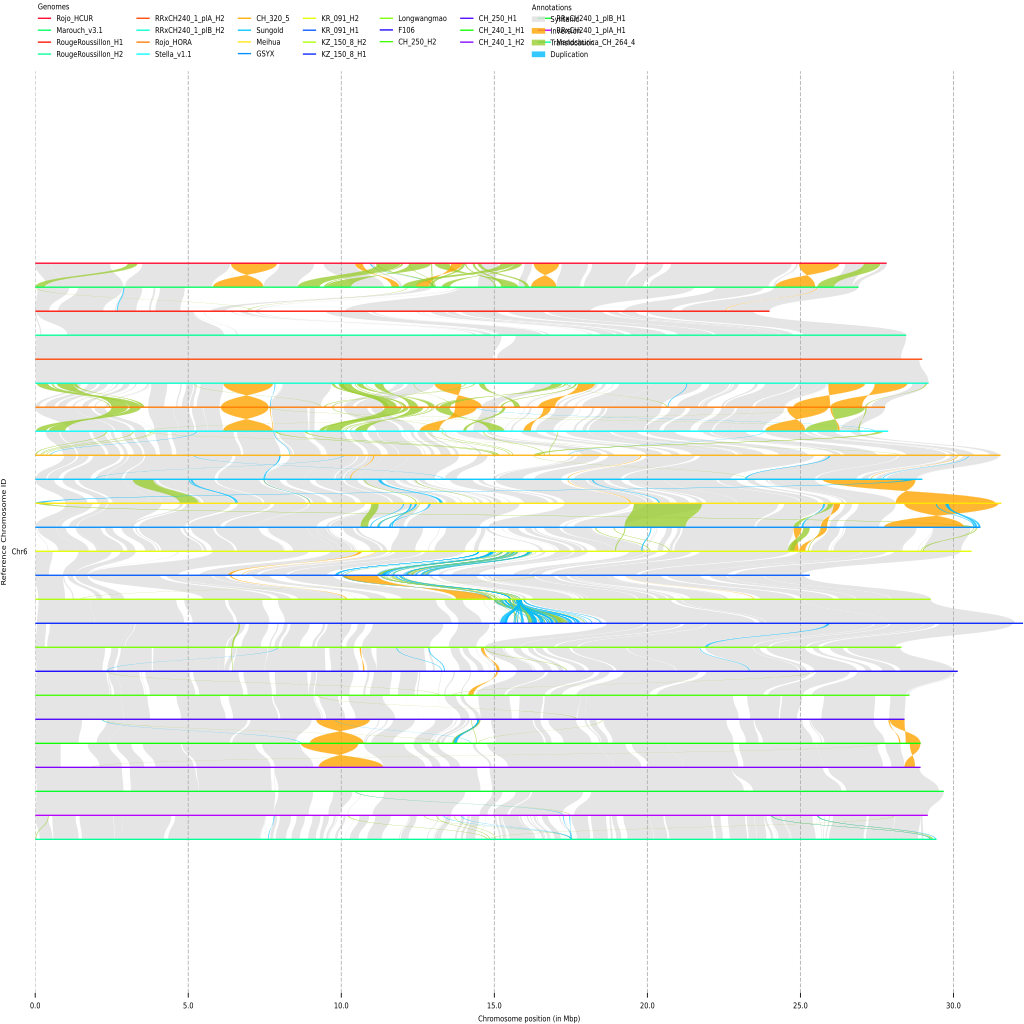


F.
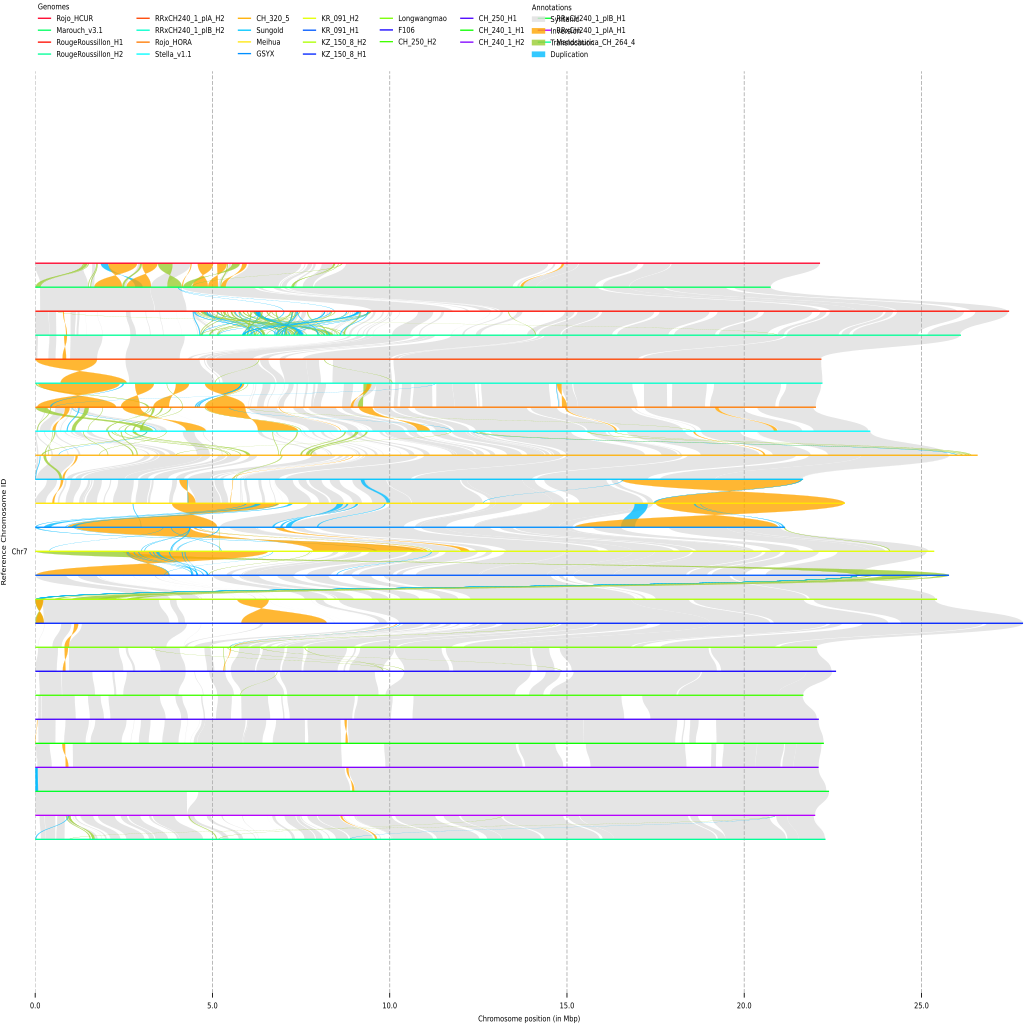


G.
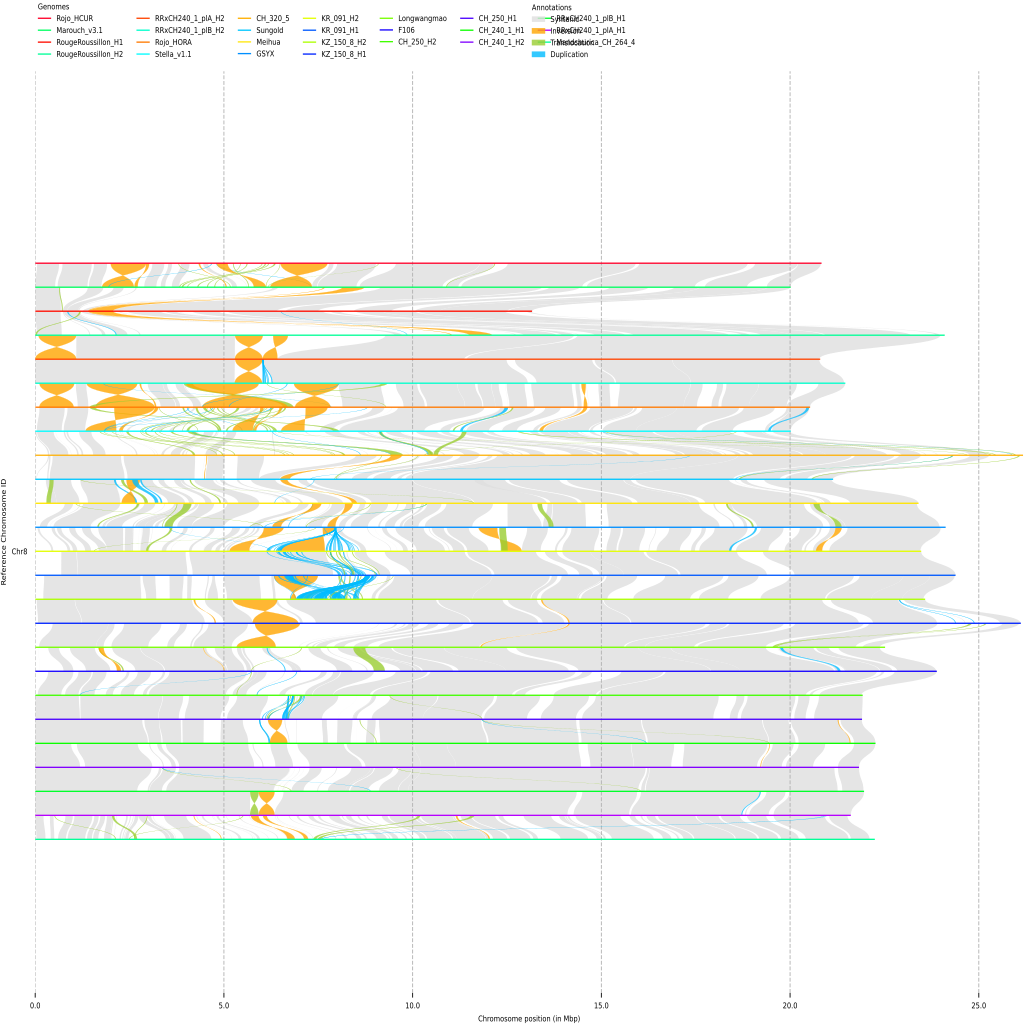


**Figure S1. Chromosome-scale synteny plots for chromosomes 2 to 8**

Pairwise synteny plots for chromosomes 2(A) to 8(G) generated using SyRI. For each chromosome, structural rearrangements including collinear regions, inversions, and translocations are shown between the reference assembly and each query assembly. Assemblies are ordered following the phylogenetic arrangement presented in Figure 1 of the main manuscript. These plots illustrate chromosome-specific patterns of structural variation and highlight assemblies exhibiting elevated levels of inversions or translocations on selected chromosomes.


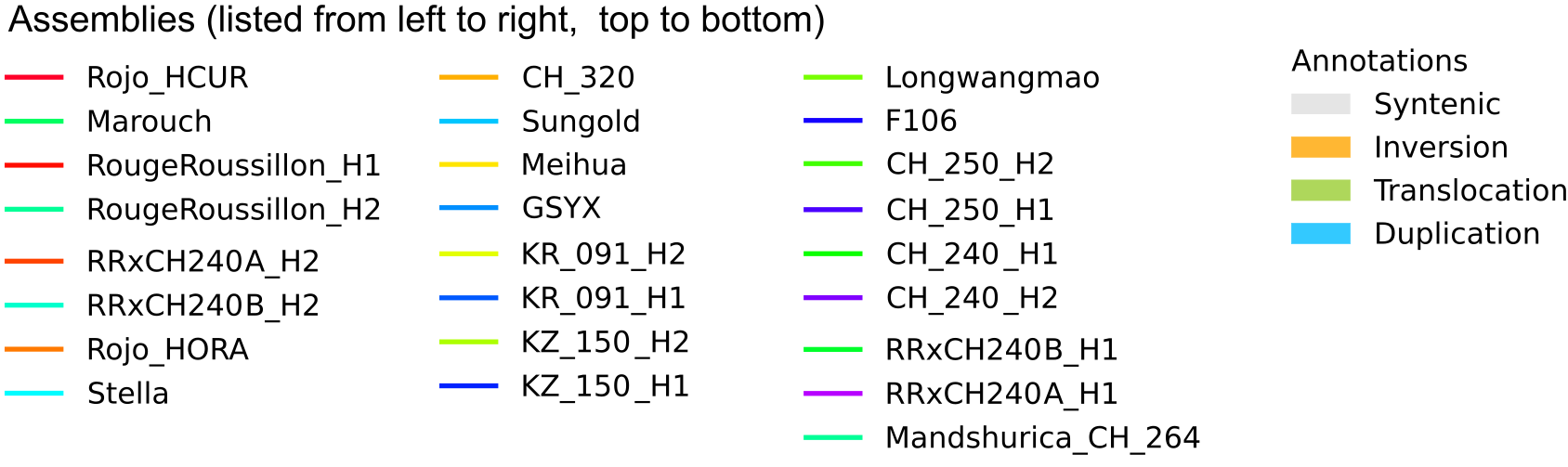


# Section 3 : Assemblies corrections

Before being used as an input to pangenome graph construction using minigraph-cactus, several assemblies were manually corrected. These corrections belongs to two main categories:

1. Inconsistent chromosome number labeling
2. Inconsistent chromosome sequence orientation

To assess the genomic relationships across assemblies, Mash distances were calculated for every chromosome against all selected scaffolds within each assembly. A Mash distance of 0 indicates identical k-mer content between two sequences, while increasing values signify greater divergence.

As depicted in the figure below, which illustrates comparisons for chromosome 1 across a subset of assemblies (represented on the T-axis), this analysis successfully identified erroneous chromosome labeling [*P. mume* tortuosa and BJFU and *P. salicina* Sanyueli_FAAS].

A.
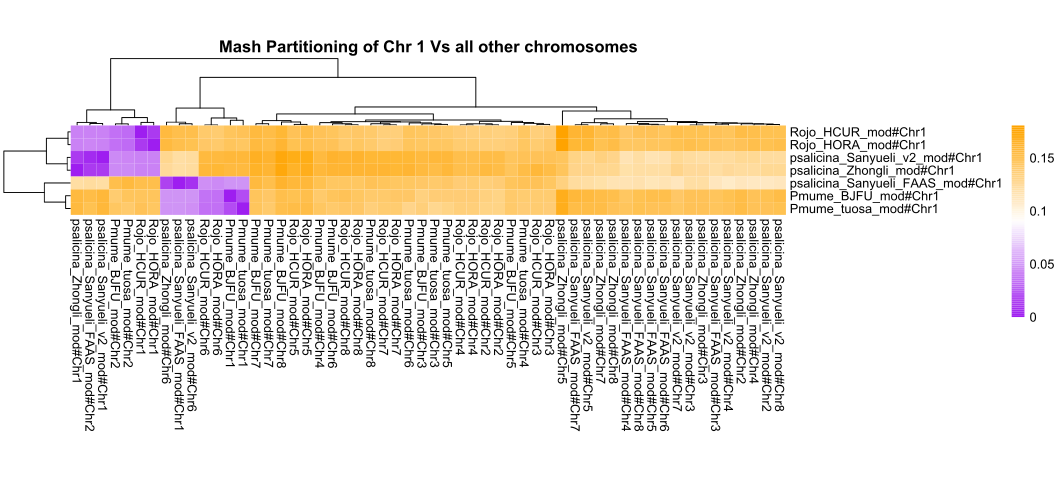


B. S


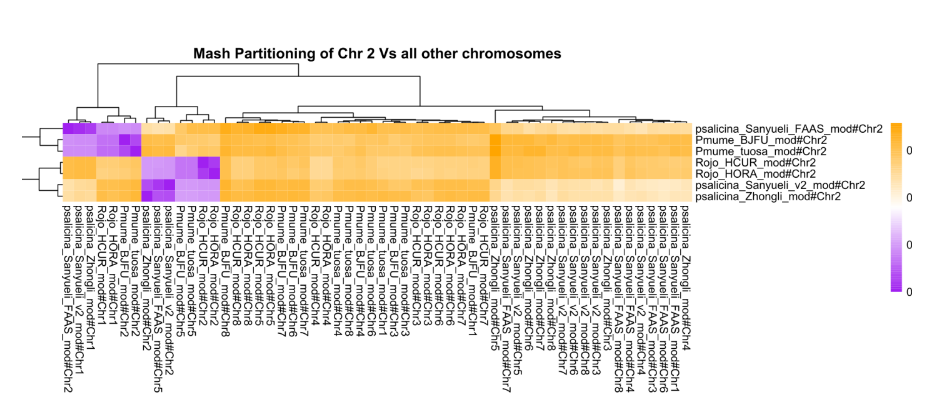


C.


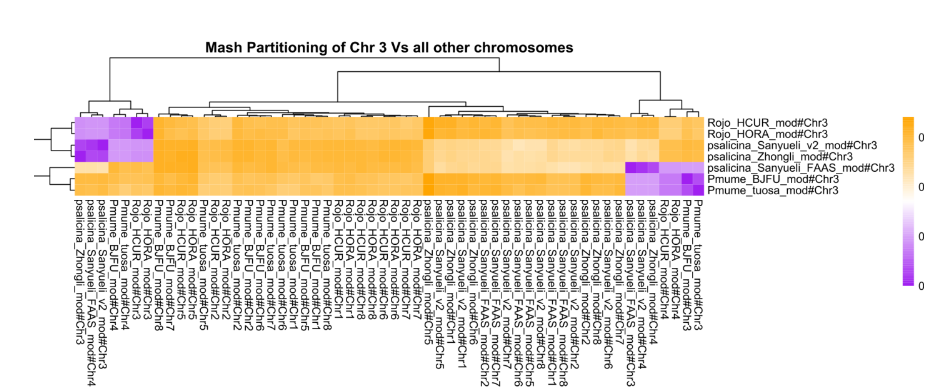


D.
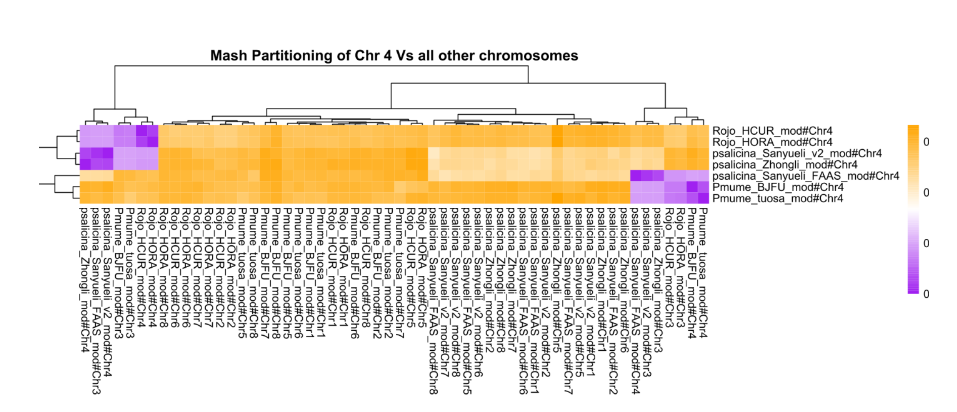


E.


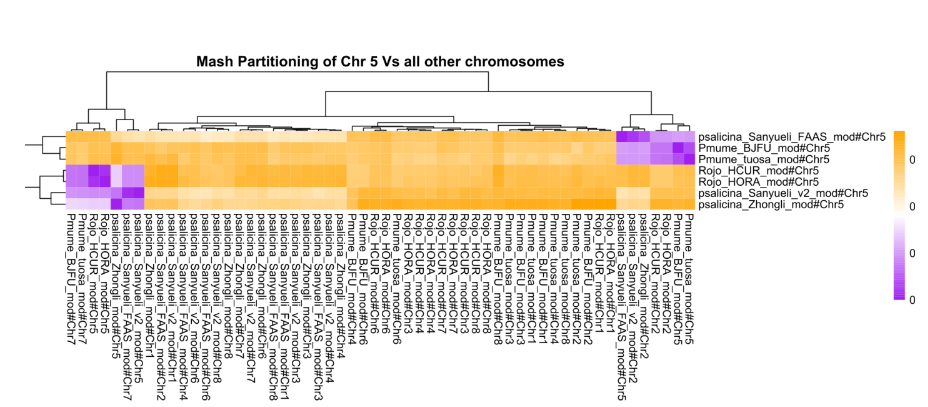


F.


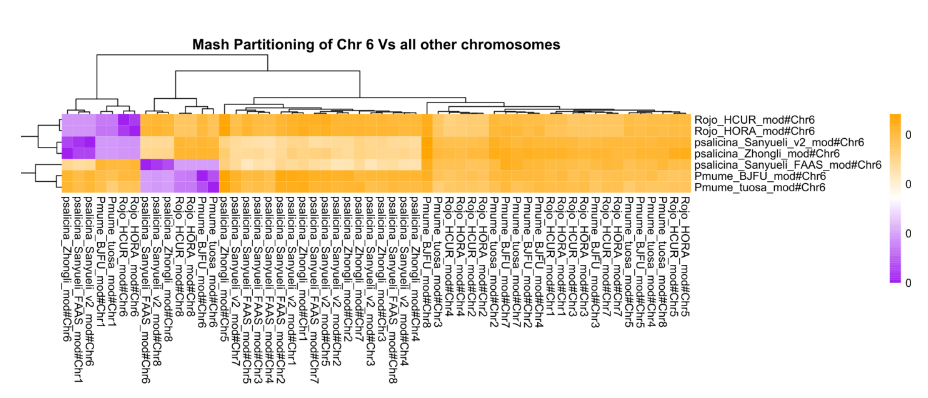


G.


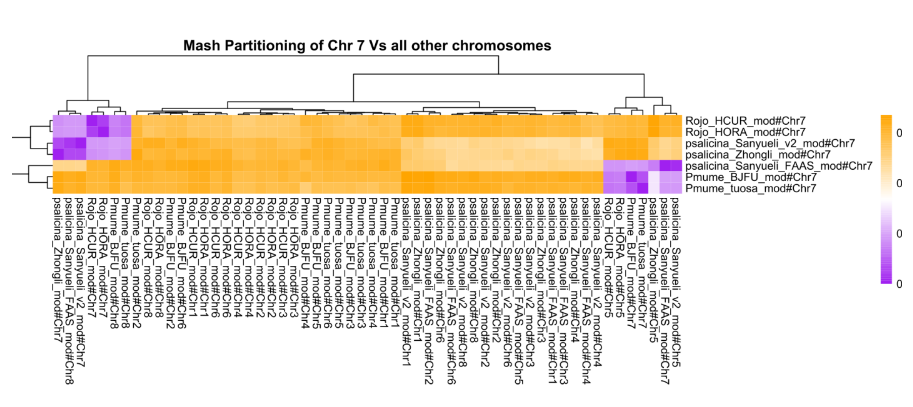


H.


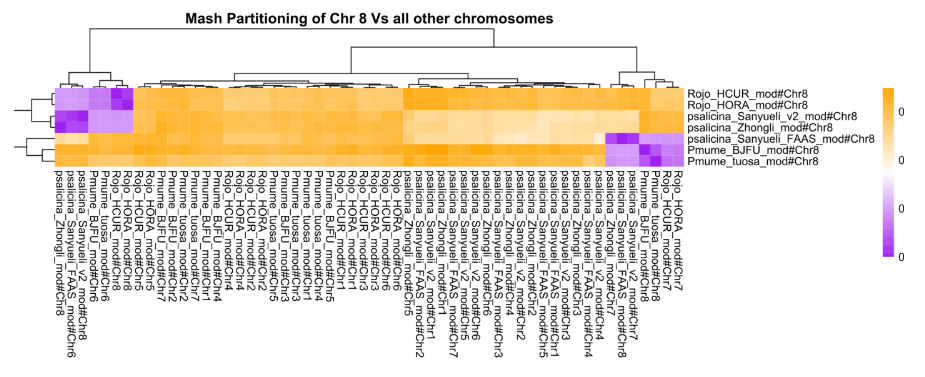


**Figure S2-A to S2-H: Inconsistencies in Chromosome 1 to 8 Labeling Across Prunus Assemblies.** Analysis of Mash distances for chromosome 1 to 8 across all retrieved Prunus assemblies revealed notable discrepancies in chromosomal labeling. A Mash distance approaching 0 indicates a high degree of similarity in k-mer content, signifying close genetic proximity.

Intriguingly, chromosome 1 of our reference assembly (Rojo_HCUR) exhibited an unexpected high proximity (low Mash distance) with chromosome 2 in several assemblies, specifically those of *P. salicina*, *P. mume* BJFU, *P. mume* tortuosa. This finding strongly suggests inconsistencies in chromosome labeling within these assemblies when compared to standard Prunus genomic nomenclature. Equivalent figures can be found for other chromosomes in Suppl Figures F1-A to F1-H.

After applying this analysis to the 8 chromosomes, we relabeled scaffold sequences in every assemblies, as described in the following text file: Suppl_Table_T1 : assemblies_sources.xlsx

For the GZYX assemblies, manual re-orientation of several chromosomes was necessary to align them with the established orientations depicted in the Genome Database for Rosaceae (GDR, www.rosaceae.org). Given the observed collinearity across *Prunus* genomes, we utilized the eight-chromosome Peach genetic architecture as our reference. The initial state of these misorientations is illustrated in the first figure, with the subsequent figure demonstrating the successful manual correction. While some smaller regions (e.g., within chromosome 2) appear to retain a reversed orientation, the predominant correct orientation of their respective chromosome sequences led us to interpret these as putative genuine inversions. Consequently, these were preserved without modification for the construction of the final graph.


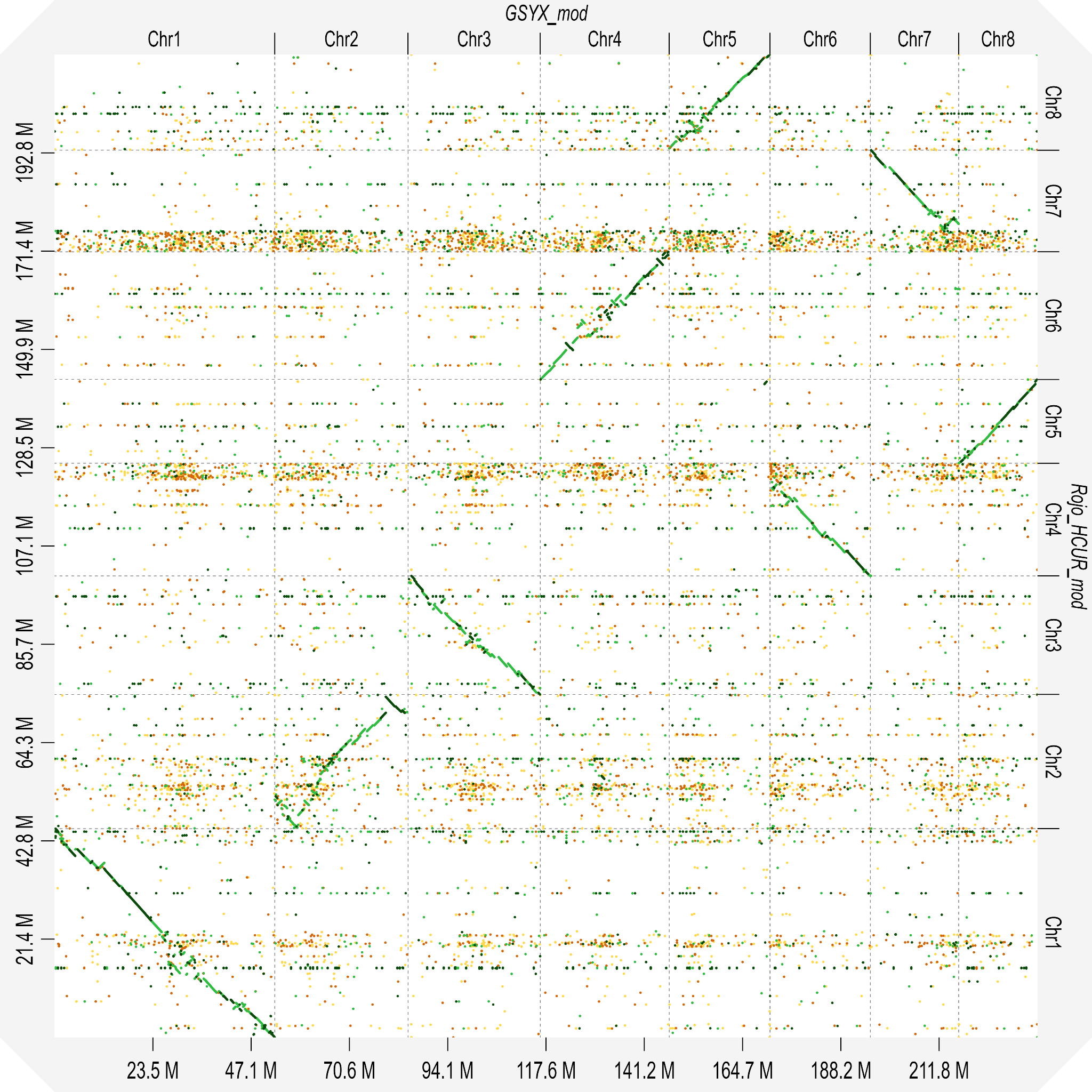


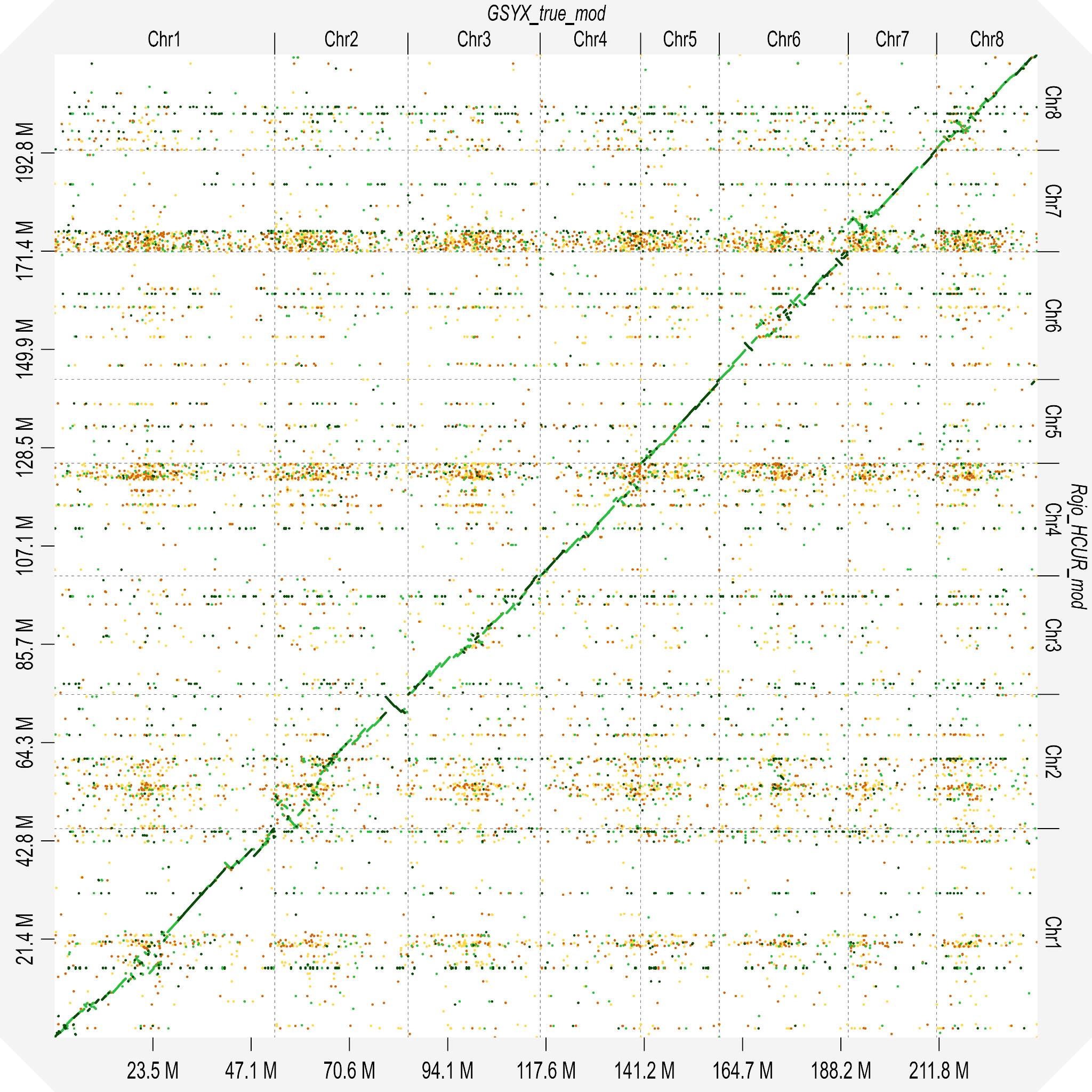


**Figure S3** : **Dotplot comparison of the GSYX haplotype before and after correction.**Dotplots generated with D-Genies show the alignment of the GSYX assembly against the reference (Rojo_HCUR) before (left) and after (right) correction. The correction process improves structural consistency and continuity, reducing noise and misalignments, particularly in complex or repetitive regions.

# Section 4: Genetic distances relative to reference assembly Rojo_HCUR

Using pantools and k-mer distances between assemblies, a genetic distance relative to reference assembly Rojo_HCUR has been estimated (Figure S3). The distance matrix can be found in Suppl Table T7.

Based on these results, we established a distance threshold of 0.03. All assemblies falling below this value were retained into the pangenome graph, as illustrated in the figure below. Specifically, *P. salicina*, *P. mume*, *P. zhengheensis* and *P. hongpingensis* were removed. Despite its proximity to this threshold, the *P. mandshurica* CH_264 assembly was also included to ensure the representation of at least one outgroup within the pangenome graph. This decision was primarily driven by the understanding that pangenome graph tools are fundamentally designed for species-centric analyses and may yield suboptimal alignments when incorporating distantly related genomes, which could impact graph quality and could generate erroneous genetic variants artifacts.


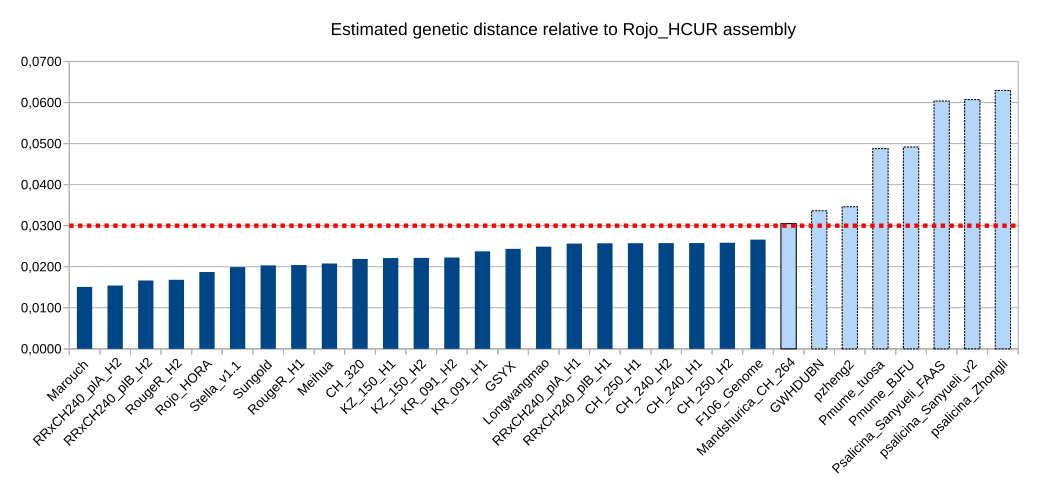


**Figure S4 : Estimated genetic distance relative to Rojo_HCUR assembly**

Assemblies used for the k-mer-based phylogenetic analysis are displayed along the X-axis, ordered by their genetic distance to the reference Rojo_HCUR. The Y-axis represents the genetic distance, with a threshold of 0.03 indicating the maximum value considered in the graph.

# Section 5: Phylogenetic tree of selected assemblies

The phylogenetic tree representing the 25 assemblies chosen for graph construction is provided below in both Newick and graphical formats. This tree exhibits a Robinson-Foulds (RF) distance of 2 when compared to the tree encompassing all assemblies, indicating a high degree of topological concordance with only two differing bipartitions. This refined tree was subsequently utilized as the guiding input for pangenome graph construction.
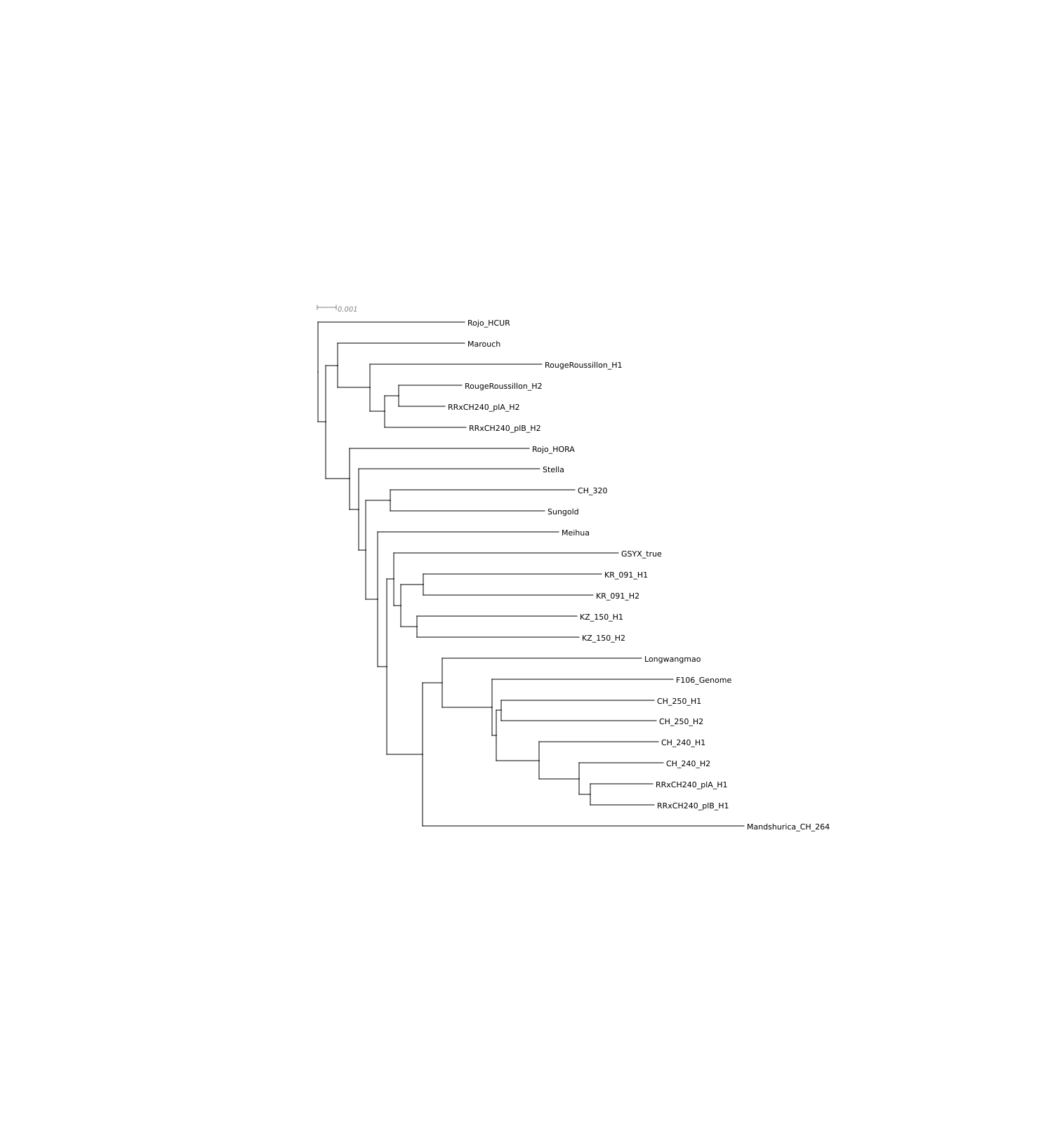


**Figure S5: Phylogenetic tree of the 25 assemblies used for pangenome graph construction**

Phylogenetic tree representing the 25 genome assemblies selected for pangenome graph construction, shown in graphical formats.

Corresponding newick tree, given as input to Minigraph-cactus. This tree and the assemblies is the input set given to minigraph-Cactus for pangenome graph construction. It is given here to allow reproducibility of its construction:

(Rojo_HCUR:0.00788576,((Marouch_v3.1:0.00681225,(RougeRoussillon_H1:0.00924739,(RRxCH240_1_plB_H2:0.00434348,(RRxCH240_1_plA_H2:0.00247215,RougeRoussillon_H2:0.00337104):0.00074349):0.00082004):0.00170963):0.00065576,(Rojo_HORA:0.00966848,(Stella_v1.1:0.00971425,((Sungold:0.00826786,CH_320_5:0.00991524):0.00134332,(Meihua:0.00973899,((GSYX_true:0.0120919,((KZ_150_8_H1:0.00860665,KZ_150_8_H2:0.0087122):0.00085192,(KR_091_H1:0.00959655,KR_091_H2:0.0091358):0.00118202):0.00039442):0.00036758,(Mandshurica_CH_264_4:0.01728973,(Longwangmao:0.01074548,(F106_Genome:0.0097333,((CH_250_H1:0.00823693,CH_250_H2:0.00832687):0.00026228,(CH_240_1_H1:0.00639755,(CH_240_1_H2:0.00452189,(RRxCH240_1_plA_H1:0.00336376,RRxCH240_1_plB_H1:0.00344785):0.00057376):0.00216333):0.00230586):0.0002406):0.00267844):0.00105252):0.00191063):0.00048916):0.0006502):0.00036991):0.00048354):0.00128998):0.00041504);

# Section 6 : Summary of Graph Metrics


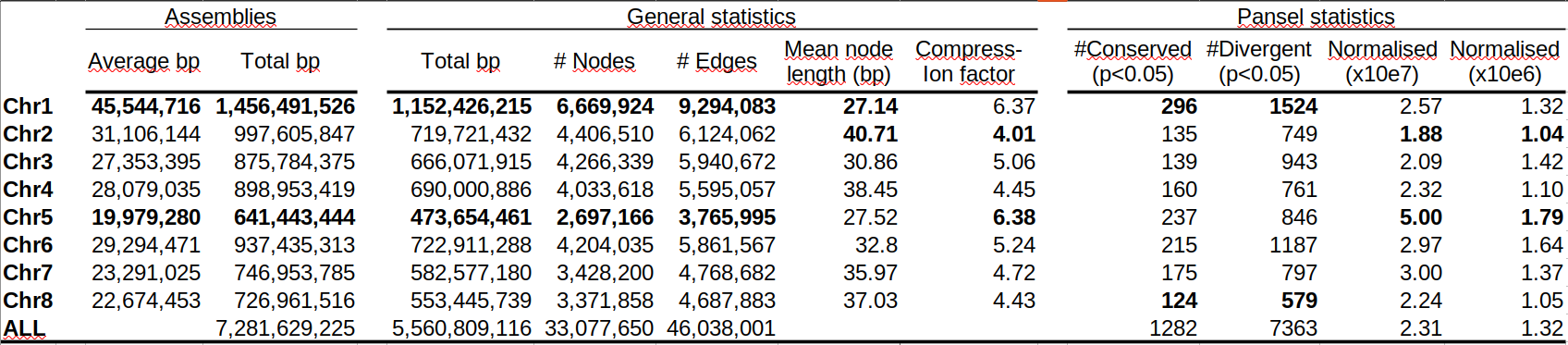
Suppl. Table S2: General statistics for the eight chromosome-level pangenome graphs.

In our pangenome graph analysis, several key metrics were employed to characterize genomic variation. The compression factor quantifies the efficiency of the graph representation, calculated as the ratio of total base pairs in the input assemblies to the total base pairs within the constructed graph. The columns labeled “Conserved regions” and “Divergent regions” report the count of genomic segments identified as significantly more conserved or divergent, respectively, relative to the entire pangenome graph and as established by the PanSel tool definition. The two rightmost columns provide a standardized measure of these regional counts by normalizing them against the corresponding chromosome length in the chosen reference assembly. The extreme values (highest and lowest) are highlighted in bold.

These statistics are complemented by several supplementary tables :

The details of Panacus values (main manuscript, figure 2) are in Suppl. Table_T2 : Ordered histgrowth in bp all chromosome.xlsx. This Excel file reports the distribution of core (red) and non-core (green) genome content along each chromosome, under two quorum conditions (0 and 1) corresponding to the Panacus execution parameters used in Figure 2 of the main manuscript. The same columns are present in all tabs, and further details on the quorum and coverage settings can be found in the Panacus documentation:<https://github.com/codialab/panacus>.

Suppl.Table_T3 : Proportion of the different genome types along the 8 chromosome.xlsx

This table provides the different proportions of core, private and accessory genome for every assembly in the graph, and for every chromosome.

Suppl.Table T7 : assemblies_distance_matrix.xlsx

This table contains the kmer distance matrix between all genome assemblies, computed from presence/absence variations across the pangenome. The values reflect the degree of shared genomic content between each pair of assemblies, as used for the clustering analysis in the main manuscript (Figure 1)

# Section 7: Complements on *P. armeniaca* transposon library generation

The eleven genome assemblies used in this study — RougeRH1, Marouch, Rojo_HCUR, Stella, KZ150H1, KR091H1, Meihua, CH240H1, CH250H1, Longwangmao, and Pman CH264 — were selected to represent the major phylogeographic groups identified within the *Prunus armeniaca* complex and related species.

Among them, the first four (RougeRH1, Marouch, Rojo_HCUR, and Stella) correspond to European cultivars. Their inclusion reflects not only their agricultural relevance but also the known genetic sub-structuring within European apricots, as demonstrated by [(Bourguiba *et al.*, 2012)](https://www.zotero.org/google-docs/?QPcNUQ), who reported distinct western and central-eastern European clusters. This intra-European divergence justified the use of multiple assemblies from this region.

In addition, two assemblies (KZ150H1 and KR091) represent Central Asian apricots, and one (Meihua) represents a Chinese *P. armeniaca*. Three others (CH240H1, CH250H1, and Longwangma*o*) correspond to *Prunus sibirica* individuals from China, and the last one *(*Pman CH264*)* belongs to *Prunus mandschurica*.

This panel was thus chosen to maximize genetic divergence while maintaining balanced representation across all known genetic groups within and around *P. armeniaca*, forming a robust foundation for pan-genomic and transposon-based comparative analyses.

To support transposon annotation and enrichment analyses across the apricot pangenome, we constructed a curated and non-redundant transposable element (TE) reference library from the 11 genome assemblies included in this study.

- Suppl.table_T8: *CatlibApricot_withoutRedundancy.classif.xlsx* This table provides the full curated list of transposable elements identified across the assemblies. For each element, it reports: sequence name, length, strand orientation, classification at multiple taxonomic levels (class, order, Wcode, sFamily), coding status, structural annotation, and a confidence index (CI). This classification enables detailed downstream analysis and comparison across TE categories.
- Suppl.data_D1: *CatlibApricot_withoutRedundancy.fa* This FASTA file contains the non-redundant nucleotide sequences of the transposable elements identified across the 11 apricot assemblies. Each sequence appears only once in the dataset, even if detected in multiple assemblies, thereby avoiding duplication and bias in enrichment analyses. The sequences are annotated with detailed metadata, including sequence name, length, strand orientation, TE classification (class, order, Wcode, sFamily), confidence index (CI), and structural or coding features.

#

# Section 8 : Supplements on SV statistics

In this section, we detail SV length distributions across assemblies and chromosomes (Figure S6) and compile comprehensive summaries of insertions, deletions, and SNPs in the supplementary data (Suppl. Table T4). Crucially, the study normalizes indel distribution and quantifies the proportional contribution of distinct phylogeographic groups to different indel size bins, providing a robust framework for evolutionary and genetic association analyses.


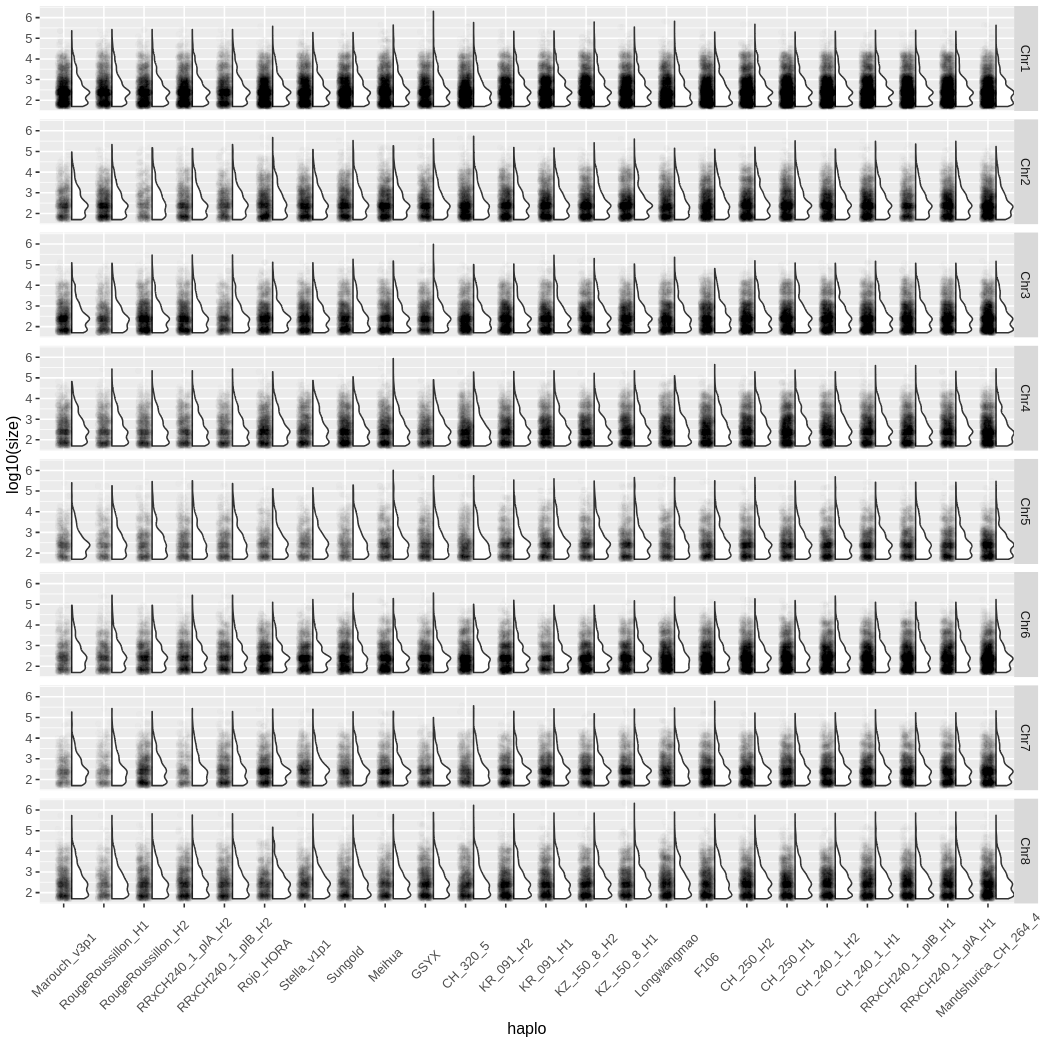


**Figure S6: Distribution of SV lengths among all chromosomes and assemblies. These indels are relative to the reference Rojo_HCUR**.

Y-axis: log10 of SV lengths. X-axis: assemblies. For each assembly and chromosome pair, the density is plotted on the right, and the corresponding values are jittered on the left.

**Suppl.table_T5 : Structural and SNP Variation Summary.xlsx**

This Excel file contains four sheets summarizing the variation landscape across the pangenome:

1. raw_summary_insertions_deletions_total_with_chr_totals — A global summary of insertions and deletions, including per-chromosome totals.
2. SNPs summary — An overview of single-nucleotide polymorphism distribution.
3. normalized_indel_bin_distribution_per_chromosome — Distribution of indels per size bin and chromosome, normalized by aligned sequence length.
4. normalized_indel_group_contribution_per_bin/chr — The proportional contribution of each phylogeographic group to indel bins, also normalized by alignment length.

#

# Section 9 : Supplements on transposons analyses


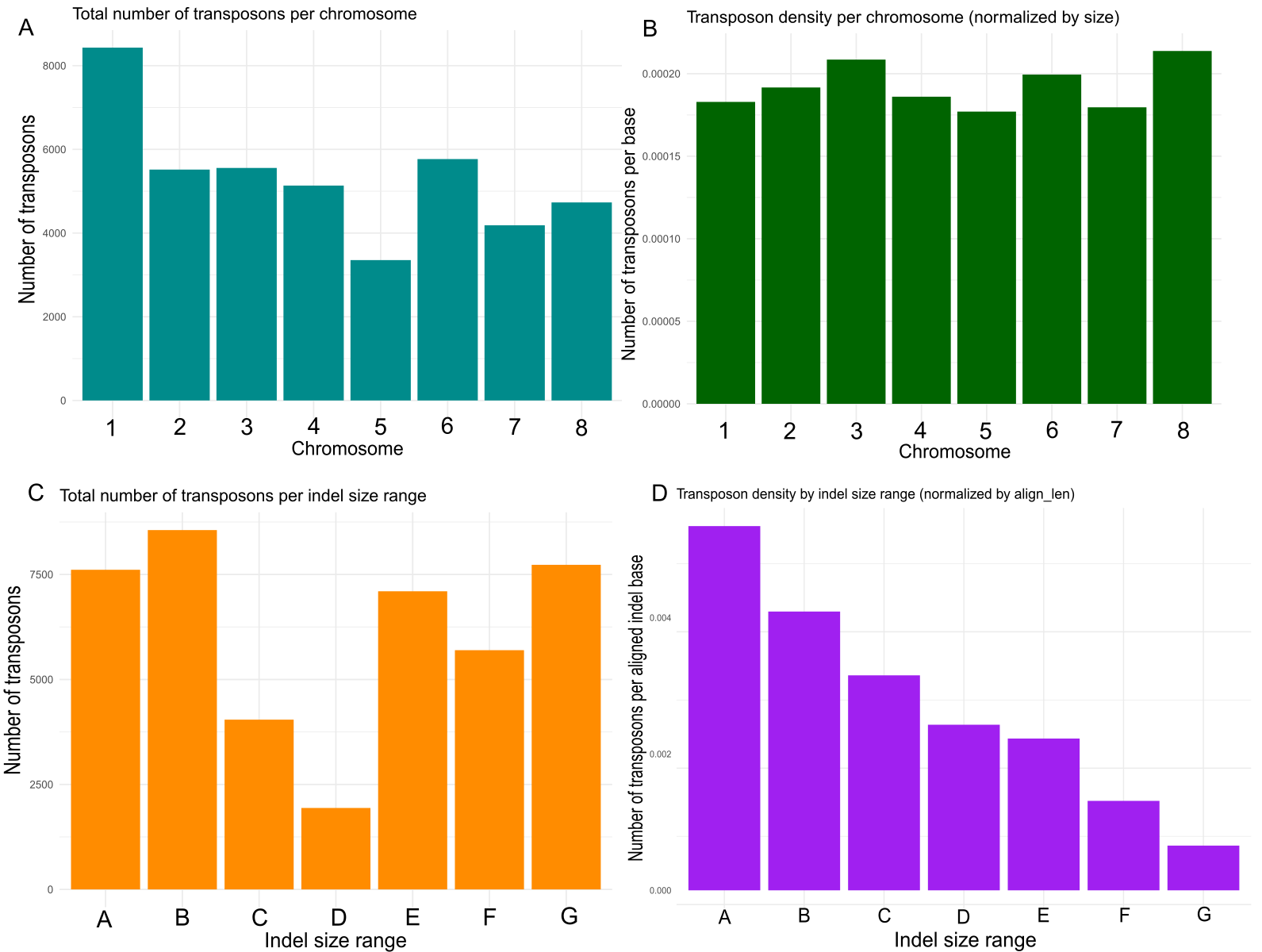


**Figure S7. Transposon distribution across chromosomes and indel size bins.**

(A) Total number of transposons per chromosome. (B) Transposon density per chromosome, normalized by chromosome size. (C) Total number of transposons per indel size bin (A to G see Figure 2E main manuscript). (D) Transposon density per indel size bin, normalized by the total aligned indel length (align_len). While raw counts reflect absolute abundance and are heavily influenced by the size of the genomic region, normalized densities effectively reveal relative enrichment, thereby highlighting distinct biological patterns across both chromosomes and indel size bins.

**Suppl.table_T6 : Transposon Distribution and Enrichment Analysis.xlsx**

This file contains six sheets focused on the relationship between indels and transposon content:

1. raw_total_transposons_by_bin_and_chromosome — Absolute counts of transposons overlapping indels, broken down by size bin and chromosome.
2. total_transposons_by_bin_and_chr_normalized — The same data normalized by the total aligned bases per category, providing a measure of density.
3. bootstrap_proportions_top5_orders_by_bins — Bootstrap-derived confidence intervals for transposon order proportions across size bins.
4. pairwise_prop_test_top5_orders_by_bins — Pairwise proportion tests to compare transposon distributions between indel size bins.
5. pairwise_prop_test_top5_orders_by_chr — The same statistical comparisons across chromosomes.
6. prop_enrichment_order_vs_other_bins — An enrichment analysis comparing specific orders to the background distribution across size bins.
7. TE_family_global_prop — Table of the proportion of the different TE_family in the # of SV overlapping TE
8. TE_order_upstream_chisq_residuals_3orders — Residuals of the chi² on haplotypes pair for 3 main orders of TE
9. TE_order_upstream_chisq_residuals_5orders — Residuals of the chi² on haplotypes pair for 5 main orders of TE
10. chi² pairwise for all haplotypes 3 and 5 orders TE —Pairwise χ² tests with Benjamini–Hochberg correction revealed that all haplotype pairs differed significantly in their TE-order composition upstream of genes

These sheets form the basis of the enrichment analyses shown in Figure 3, highlighting how transposon density varies according to indel length and chromosomal location.

**Indel Size bins**

In raw counts, mid-sized bins such as 200–400 bp (8,557 transposons) and 800–1,800 bp (7,100 transposons) have the highest number of insertions, suggesting a concentration of transposons in these indel classes (figure S7A). However, after normalization by the total aligned bases (figure S7B), the 50–200 bp bin becomes the most enriched, with the highest density (~0.00534 transposons per aligned base), indicating that short indels are disproportionately loaded with transposons. Conversely, longer indels (e.g., 5,000–100,000 bp) show lower densities (~6.56 × 10⁻⁴), despite having high raw counts, suggesting a more dispersed insertion pattern.

**Transposon content per chromosomes**

When looking at raw transposon counts per chromosome (figure S7C), chr1 leads with 8,431 insertions, followed by chr6 (5,768) and chr2 (5,517). These values largely reflect the size of the chromosomes. However, normalization by chromosome length reveals a different trend (figure S7D): chr8 shows the highest transposon density (~2.14 × 10⁻⁴), followed by chr3 (~2.08 × 10⁻⁴) and chr6 (~1.99 × 10⁻⁴). In contrast, chr5 has both the lowest raw count (3,354) and the lowest normalized density (~1.77 × 10⁻⁴), suggesting that it is overall less enriched in transposons.

**Summary comparison**

Raw counts reflect absolute abundance and are strongly influenced by region size.

Normalized densities reveal relative enrichment, highlighting biologically meaningful patterns that are not apparent from raw counts alone. For indels, short bins are the most enriched despite smaller absolute counts. For chromosomes, medium-sized ones (like chr8 and chr3) show the highest density, whereas chr5 stands out as both short and less enriched.

**Transposon type classification**

The classification and nomenclature of transposon types used in this study adhere to the definitions outlined in Figure 1 of [(Wicker *et al.*, 2007)](https://www.zotero.org/google-docs/?jzkziN). For composite elements, such as TIR|LTR, the pipeline detects characteristic patterns from either the TIR or the LTR family, this behavior is common for all elements composed of only two mixed families. If an element exhibits characteristics from more than three families, it is designated as Unclassified.

**The difference in the proportion profile of transposable element (TE) orders between the haplotypes**

**
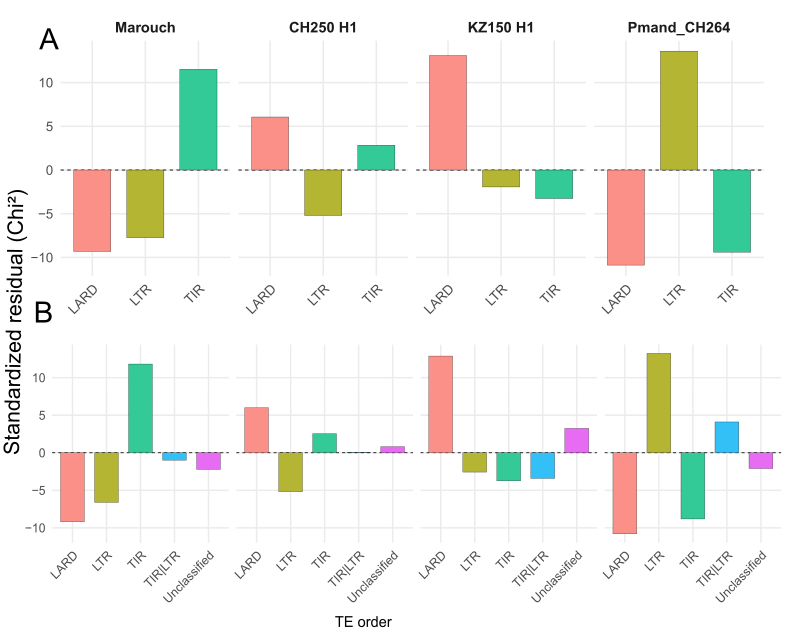
**

**Figure S8 Haplotype-specific proportions of TE orders upstream of genes.**

(A) Proportion profile for three major TE orders (LARD, LTR, TIR).
(B) Proportion profile for five TE orders (LARD, LTR, TIR, TIR|LTR, Unclassified).

Bars show standardized residuals from χ² tests comparing the observed counts of SV-associated TEs per haplotype and TE order to expectations under a homogeneous composition across haplotypes. Positive residuals indicate enrichment of a given order in a given haplotype, whereas negative residuals indicate depletion. Values with |residual| ≳ 2 correspond to strong deviations from the overall expectation. Panel A summarizes the enrichment patterns for three major orders (LARD, LTR, TIR), and panel B extends this analysis to five orders, adding TIR|LTR and Unclassified, highlighting distinct TE-order signatures for each haplotype upstream of genes.

The composition of transposable element (TE) orders varied significantly among the different haplotypes in the regions upstream of genes (Figure S8 A). Using a simplified three-order model (LARD, LTR, and TIR), we observed distinct patterns:

- Marouch was strongly enriched in TIR elements (residual ≈ +11.5), showing a marked depletion of both LARD and LTR elements (resid ≈ –9.3 and –7.7, respectively).
- Pman_CH264 exhibited a strong enrichment in LTR elements (resid ≈ +13.6) and a corresponding depletion of LARD and TIR (resid ≈ –10.9 and –9.4).
- KZ150 H1 displayed the opposite trend, with a pronounced enrichment in LARD (resid ≈ +13.1) and a depletion in TIR.
- Finally, CH250 H1 showed only moderate deviations, with LARD and TIR slightly enriched and LTR depleted.

Extending the analysis to five distinct TE orders (LARD, LTR, TIR, TIR|LTR, Unclassified) revealed patterns consistent with the initial findings (Figure S8 B). Specifically:

- **Marouch** remained enriched in TIR elements while depleted in LARD/LTR
- Pman_CH264 stayed strongly enriched in LTR elements and depleted in LARD and TIR.
- KZ150 H1 combined a strong enrichment of LARD with a mild excess of Unclassified elements, showing depletion in both TIR and TIR|LTR.
- CH250 H1 again demonstrated only modest deviations across all TE orders.

Collectively, these results clearly indicate that each haplotype possesses a unique and distinct TE-order signature upstream of genes, confirming that no two haplotypes share comparable TE-order profiles.

# Section 10 : Filtering of reads after mapping

**Limitations of Pangenome Graph Tools in Handling SAM Flags**

We observed that current pangenome graph tools, specifically VG tools (v1.57), do not fully support all SAM flags. A notable limitation was identified in the 'vg surject' command; when projecting to references using a GAM file (e.g., from 'vg giraffe' output), information regarding read pairs is not retained. Consequently, pair-based filtering functionality is currently incomplete.

Furthermore, we did not observe any SAM flags associated with secondary or supplementary alignments in the output of 'vg giraffe'. While an option exists to output secondary alignments, preliminary testing indicated it may be non-functional, as its activation drastically reduced the quantity of mapped reads. It is however important to note these observations are specific to the version of VG tools used in this study, and ongoing active development may lead to rapid improvements in their functionality.

To illustrate the aforementioned issue, we performed a comparative analysis of Minimap2 and VG Giraffe using a small subset of samples. This comparison involved different FLAG-based filtering strategies applied to their respective mapping outputs.

- For Minimap2's BAM output, secondary and supplementary alignments were discarded (consistent with their lack of handling by VG tools), and unmapped reads were also excluded. Read pairs with only one mapped mate were retained, achieved by applying the flag filters '-f 4 -F 2304'.
- Similarly, for the VG Giraffe GAM output, subsequently processed by VG Surject to produce a BAM file, secondary and supplementary alignments were discarded (as VG would not set these flags). Unmapped reads were also removed, and read pairs with a single mapped mate were retained using the flag filter '-f 4 -F 2304'.

While the above limitations exist, it is possible to obtain the desired SAM flags by directly projecting during a VG giraffe run onto a pre-selected set of references. This process will lead to proper inclusion of these flags in the resulting BAM output. However, this approach requires executing the 'vg giraffe' command twice, as it currently lacks the functionality to output two distinct formats within a single run. Such a dual execution would incur prohibitive CPU costs, leading us to restrict this particular analysis to a subset of 50 short-read samples.

This small comparative analysis is based on six distinct conditions:

- Minimap2, with no multimapping, requires both mates to be mapped.
- Minimap2, with no multimapping, adhering to the "pair properly mapped" definition (which is software-dependent).
- Giraffe, with projection to all assemblies (ALLREFS), requiring both mates to be mapped.
- Giraffe, with projection to all assemblies (ALLREFS), adhering to the "pair properly mapped" definition.
- Giraffe, with projection exclusively to Rojo_HCUR (1REF), requiring both mates to be mapped.
- Giraffe, with projection exclusively to Rojo_HCUR (1REF), adhering to the "pair properly mapped" definition.

The outcomes of this comparative analysis are presented in the table and plot below. The corresponding SAM flags for each condition are detailed in the legend.


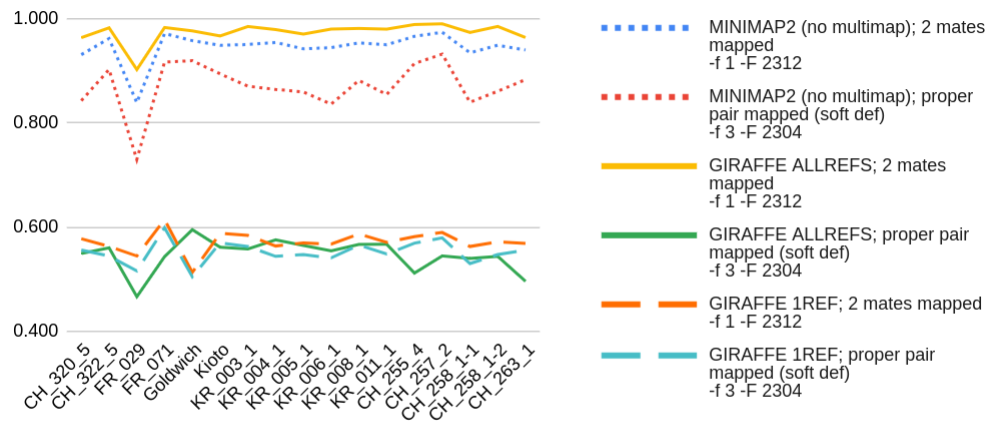


**Figure S9 : Comparative read-pair alignment performance across aligners, reference configurations and flag filters.** Blue dotted line — Minimap2, multimapping suppressed, pairs with both mates mapped (-f 1 -F 2312).
 Red dotted line — Minimap2, multimapping suppressed, pairs flagged as properly mapped (-f 3 -F 2304, definition software-dependent).
 Yellow solid line — VG Giraffe projected to all assemblies (ALLREFS), pairs with both mates mapped (-f 1 -F 2312).
 Green solid line — VG Giraffe (ALLREFS), pairs flagged as properly mapped (-f 3 -F 2304).
 Orange dashed line — VG Giraffe projected exclusively to Rojo_HCUR (1REF), pairs with both mates mapped (-f 1 -F 2312).
 Light-blue dashed line — VG Giraffe (1REF), pairs flagged as properly mapped (-f 3 -F 2304).

Note : Counts obtained with -f 1 -F 2312 reflect read pairs in which both mates are mapped and all secondary/supplementary alignments are removed. Counts obtained with -f 3 -F 2304 correspond to the SAM flag “properly mapped” category; this classification is aligner-specific and may include additional internal consistency checks.

Regarding the analysis of unpaired reads, our findings consistently demonstrate that graph-based mapping yields a higher proportion of paired alignments compared to conventional methods when projecting across all available assemblies (as evidenced by the yellow line versus the blue line). However, a significant limitation was observed with 'vg giraffe' under single-reference projection conditions (1REF), where nearly half of the read pairs failed to achieve concordant mapping. Even more unexpectedly, projection to the Rojo_HCUR reference (orange dashed line) resulted in only 50% to 60% of pairs remaining mapped, a proportion substantially lower than what was obtained with Minimap2.

These observations suggest that the current version of VG surject is unadapted to robust handling of BAM-like files and their associated flag-based filtering. This presents a bottleneck in results analysis, as the only alternative is the GAM binary format output by VG giraffe : a binary file that involves advanced programming expertise for effective exploitation.

# Section 11 : MAPQ scores distribution following mapping

MAPQ scores are designed to distinguish high-quality from low-quality mappings. However, the formula used to compute this value is author and software dependent. Thus, direct comparison using thresholds is not valid. See these [explanations](https://www.acgt.me/blog/2014/12/16/understanding-mapq-scores-in-sam-files-does-37-42) (https://www.acgt.me/blog/2014/12/16/understanding-mapq-scores-in-sam-files-does-37-42) for an oversight from other authors on this interesting point.

Moreover, as shown in the following figure, the MAPQ distribution generated by Minimap2 and VG Giraffe appears quite different. Strikingly, MAPQ peaks seem to appear every 15 MAPQ units on average, which we hypothesised to be an artifact of normalization by read length (reads from the 322 mapped accessions are 150 bp long, on average). Note that this pattern is absent from Minimap2 MAPQ scores.


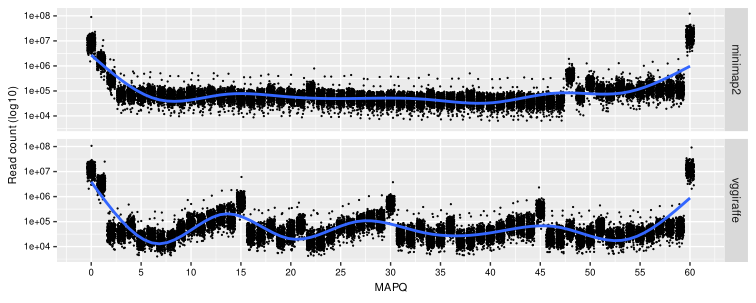


**Figure S10: Distribution of MAPQ scores, using all mappings from all accessions.** For each MAPQ value, read count per accession is jittered. The blue line corresponds to the fitted Generalized Additive model (GAM) with smoothing parameters selected by restricted Marginal Likelihood (REML) estimation.

#

# Section 12 : Complements on the evaluation of mapping accuracy

Suppl. Table S3 reports the recall observed for each accession when remapped to the depleted graphs. Recall includes both the forward and reverse mate counts, and as a consequence cases where only one mate is remapped.

| Haplotype | **Recall** |
| --- | --- |
| CH240_H1 | 0.9830503271 |
| CH240_H2 | 0.9726686593 |
| KZ150_H1 | 0.9700890098 |
| KZ150_H2 | 0.9694891647 |
| Marouch | 0.9614045288 |
| **MEAN** | 0.9713403379 |

Suppl. Table S3 : Recall of simulated reads after remapping to depleted graphs.

Figure S11 provides a detailed view of the full positional shift distributions obtained when mapping simulated paired-end reads to haplotype-depleted pangenome graphs. Only reads where both mates were remapped were considered for this result. Panel A shows the distribution of mapping offsets (Δ) for the two mates of the simulated paired reads, Read 1 (R1) and Read 2 (R2), across all tested haplotypes. In all cases, a dominant peak centered at Δ = 0 is observed, indicating a high proportion of reads mapped exactly to their true position. The logarithmic scaling of read counts further reveals the presence of long but rare cases of long shifts (below -200bp or above 200bp). Manual investigation highlighted some cases of reads holding low complexity sequences (simple repeats, or long chunks of repeated bases). It is notoriously known that such patterns are likely to align with different, distant, chromosome loci by chance. Panel B displays the empirical cumulative distribution functions (ECDFs) of the absolute positional error |Δ| over the full error range. For all haplotypes, R1 and R2 exhibit similar overall trends, with a rapid initial increase corresponding to accurately placed reads until 85% are placed for a delta of 30 bp, followed by a slower accumulation driven by rare large-offset events. While R2 consistently shows a broader error distribution than R1 at small |Δ| values, both read mates ultimately converge toward identical cumulative behavior over the full range, indicating that large positional errors remain rare events. Notably, read simulations with VG did not involve quality simulation (e.g. read associated Phred scores), and as a consequence this bias may be inherent to the mapper that we used and may be corrected in future versions.


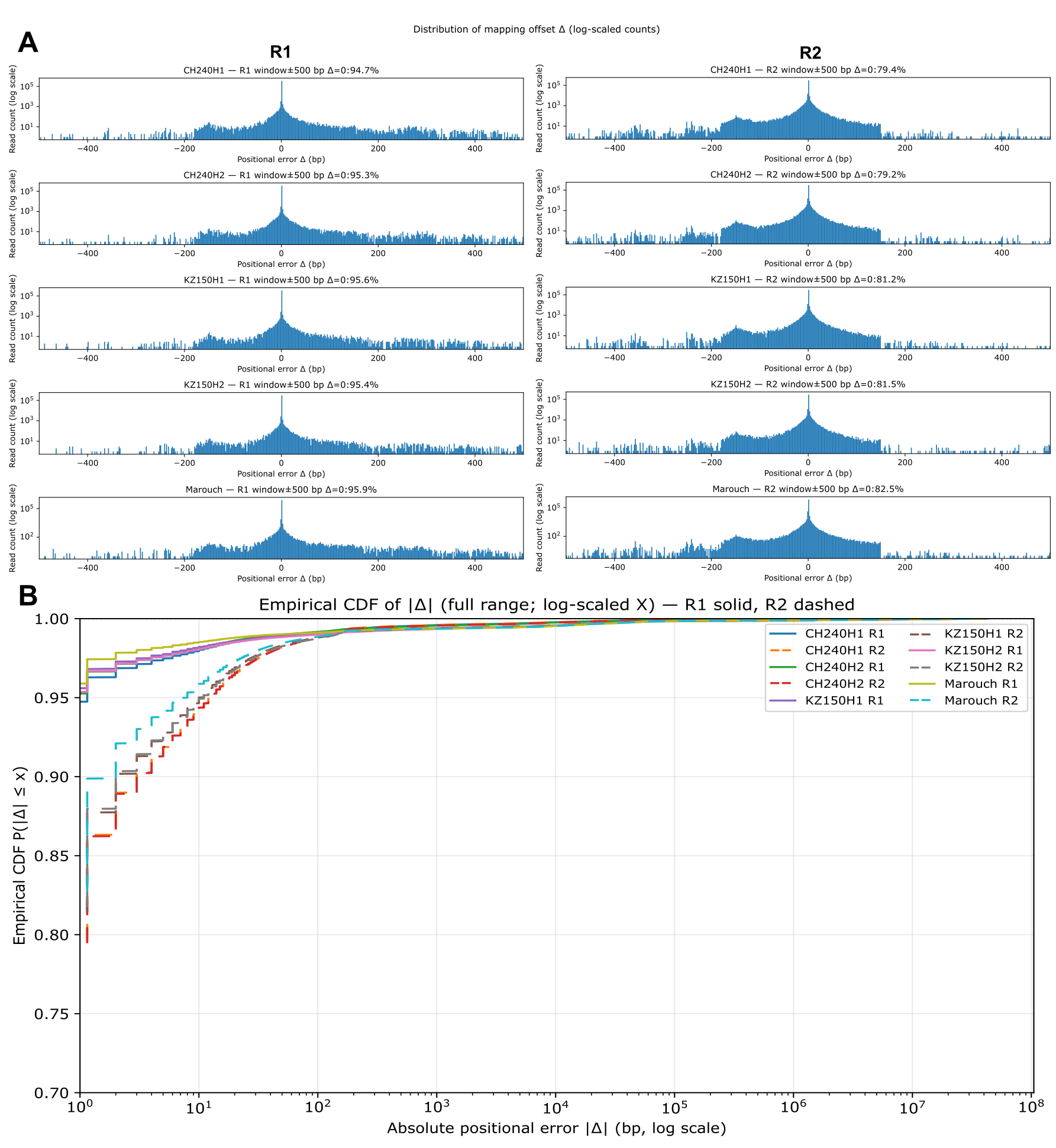
**Figure S11 | Full distribution of positional errors for simulated reads mapped to haplotype-depleted pangenome graphs**

**A.** Distribution of mapping offset Δ for simulated paired-end reads mapped to haplotype-depleted pangenome graphs. Histograms show the count of reads (Y-axis, log10 scaled) as a function of the positional shift Δ ( X axis, in base pairs) between the true coordinate of the simulated read and the observed coordinate after remapping. Read 1 (R1) and Read 2 (R2) are shown separately. This representation highlights both the dominant peak at Δ = 0 and the full range of positional offsets observed across all datasets. **B.** Empirical cumulative distribution function (ECDF) of the absolute positional error |Δ| computed over the full error range for simulated paired-end reads. The X-axis shows |Δ| in base pairs on a logarithmic scale, while the Y-axis represents the cumulative fraction of reads with |Δ| ≤ x. Curves are shown separately for Read 1 (R1) and Read 2 (R2), allowing direct comparison of their global error distributions without conditional restriction.

# Section 13 : Data relative to the DAM Genomic Region

For the selection of large indels highlighted in Figure 5 of the main manuscript, the coordinates of the corresponding graph loops can be found in Suppl.table_T9. It contains 2 sheets.

- - Sheet 1: “positions_in_DAM_loops_assembly” contains said positions.
  - Sheet 2: ”transposon_match_per_loop” : For each loop, we report the transposon matches.

To confirm the presence of the DAM region in the *Prunus mandshurica* CH264 assembly, despite its reported absence in the main manuscript, we performed BLAST searches on the raw reads. Results are available in Supplementary Table T10: Blast_DAM1_to_6_ch_264_4.xlsx.

#

# Section 14 : Computational cost of mapping

While VG tools enable a higher proportion of reads to be mapped, this advantage comes with a substantial computational burden. Across the various mapping configurations tested (detailed in section 10), the CPU cost ranged significantly. Mapping projected to the Rojo_HCUR reference incurred an average of 50 CPU hours per sample, whereas projection to all assemblies escalated the average to 175 CPU hours per sample.


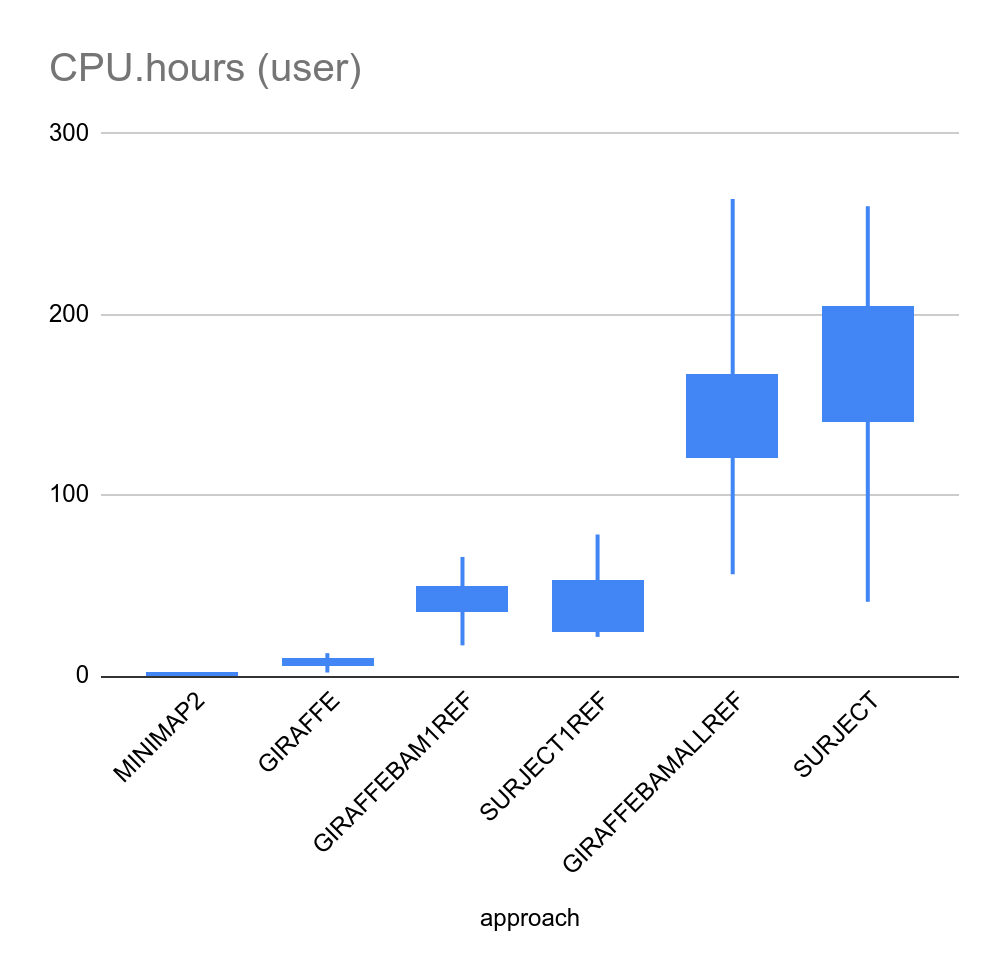

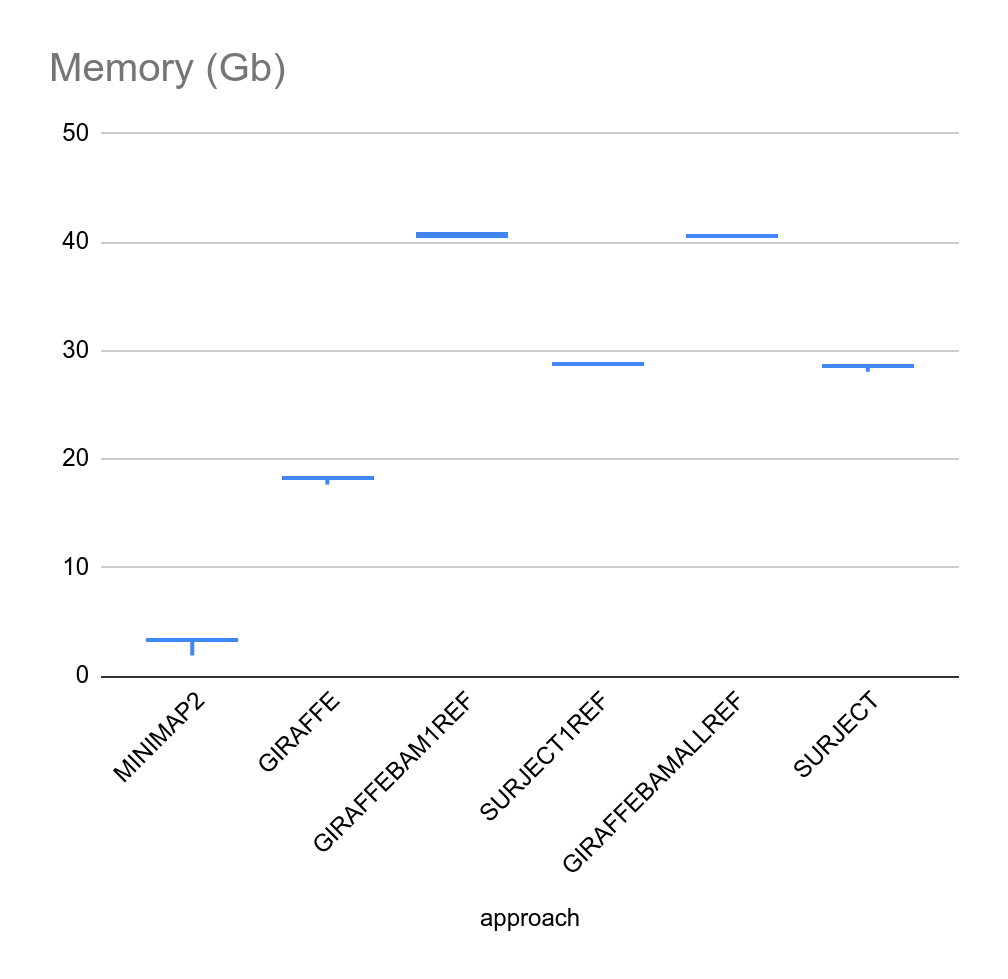


**Figure S12 : CPU and memory cost of the different mapping method (minimap and VG) and their different steps.** Minimap2 corresponds to linear mapping. Giraffe indicates direct graph mapping with internal BAM output and implicit surjection. GiraffeBAM1ref and GiraffeBAMallref use vg giraffe with internal BAM projection to a single reference path (Rojo_HCUR) or all available paths, respectively. Surject1ref and Surjectallref separate the pipeline, with vg giraffe producing GAM files followed by explicit surjection using vg surject to one or all reference paths

Memory costs remained stable, as they are related to the size of the analyzed pangenome and associated indexes.
